# Dendritic Cell‐Mediated Cross‐Priming by a Bispecific Neutralizing Antibody Boosts Cytotoxic T Cell Responses and Protects Mice against SARS‐CoV‐2

**DOI:** 10.1002/advs.202304818

**Published:** 2023-10-20

**Authors:** Rodrigo Lázaro‐Gorines, Patricia Pérez, Ignacio Heras‐Murillo, Irene Adán‐Barrientos, Guillermo Albericio, David Astorgano, Sara Flores, Joanna Luczkowiak, Nuria Labiod, Seandean L. Harwood, Alejandro Segura‐Tudela, Laura Rubio‐Pérez, Yudhi Nugraha, Xiaoran Shang, Yuxing Li, Carlos Alfonso, Kaylin A. Adipietro, Dinendra L. Abeyawardhane, Rocío Navarro, Marta Compte, Wenbo Yu, Alexander D. MacKerell, Laura Sanz, David J. Weber, Francisco J. Blanco, Mariano Esteban, Edwin Pozharski, Raquel Godoy‐Ruiz, Inés G. Muñoz, Rafael Delgado, David Sancho, Juan García‐Arriaza, Luis Álvarez‐Vallina

**Affiliations:** ^1^ Cancer Immunotherapy Unit (UNICA) Department of Immunology Hospital Universitario 12 de Octubre Madrid 28041 Spain; ^2^ Immuno‐Oncology and Immunotherapy Group Instituto de Investigación Sanitaria 12 de Octubre (imas12) Madrid 28041 Spain; ^3^ H12O‐CNIO Cancer Immunotherapy Clinical Research Unit Centro Nacional de Investigaciones Oncológicas (CNIO) Madrid 28029 Spain; ^4^ Department of Molecular and Cellular Biology Centro Nacional de Biotecnología (CNB) Consejo Superior de Investigaciones Científicas (CSIC) Madrid 28049 Spain; ^5^ Centro de Investigación Biomédica en Red de Enfermedades Infecciosas (CIBERINFEC) Madrid 28029 Spain; ^6^ Immunobiology lab Centro Nacional de Investigaciones Cardiovasculares (CNIC) Madrid 28029 Spain; ^7^ Virology and HIV/AIDS Group Instituto de Investigación Sanitaria 12 de Octubre (imas12) Madrid 28041 Spain; ^8^ Department of Molecular Biology and Genetics – Protein Science Aarhus University Aarhus 80000 Denmark; ^9^ Chair for Immunology UFV/Merck Universidad Francisco de Vitoria (UFV) Pozuelo de Alarcón Madrid 28223 Spain; ^10^ Protein Crystallography Unit Structural Biology Programme Centro Nacional de Investigaciones Oncológicas (CNIO) Madrid 28029 Spain; ^11^ Institute for Bioscience and Biotechnology Research University of Maryland Rockville MD 20850 USA; ^12^ Department of Microbiology and Immunology University of Maryland School of Medicine Baltimore MD 21201 USA; ^13^ The Center for Biomolecular Therapeutics Rockville MD 20850 USA; ^14^ Centro de Investigaciones Biológicas Margarita Salas (CIB) Consejo Superior de Investigaciones Científicas (CSIC) Madrid 28040 Spain; ^15^ Biochemistry and Molecular Biology University of Maryland School of Medicine Baltimore MD 21201 USA; ^16^ Department of Antibody Engineering Leadartis SL Tres Cantos Madrid 28002 Spain; ^17^ Computer Aided Drug Design Center Department of Pharmaceutical Sciences University of Maryland School of Pharmacy Baltimore MD 21201 USA; ^18^ Center for Biomolecular Therapeutics (CBT) University of Maryland School of Medicine Baltimore MD 21201 USA; ^19^ Molecular Immunology Unit Hospital Universitario Puerta de Hierro Majadahonda Majadahonda Madrid 28220 Spain; ^20^ Department of Microbiology Hospital Universitario 12 de Octubre Madrid 28041 Spain; ^21^ Department of Medicine Universidad Complutense de Madrid Madrid 28040 Spain

**Keywords:** bispecific trimerbody, cross‐priming, CTL responses, dendritic cells, neutralizing antibody, SARS‐CoV‐2

## Abstract

Administration of neutralizing antibodies (nAbs) has proved to be effective by providing immediate protection against SARS‐CoV‐2. However, dual strategies combining virus neutralization and immune response stimulation to enhance specific cytotoxic T cell responses, such as dendritic cell (DC) cross‐priming, represent a promising field but have not yet been explored. Here, a broadly nAb, TN^T^, are first generated by grafting an anti‐RBD biparatopic tandem nanobody onto a trimerbody scaffold. Cryo‐EM data show that the TN^T^ structure allows simultaneous binding to all six RBD epitopes, demonstrating a high‐avidity neutralizing interaction. Then, by C‐terminal fusion of an anti‐DNGR‐1 scFv to TN^T^, the bispecific trimerbody TN^T^DNGR‐1 is generated to target neutralized virions to type 1 conventional DCs (cDC1s) and promote T cell cross‐priming. Therapeutic administration of TN^T^DNGR‐1, but not TN^T^, protects K18‐hACE2 mice from a lethal SARS‐CoV‐2 infection, boosting virus‐specific humoral responses and CD8^+^ T cell responses. These results further strengthen the central role of interactions with immune cells in the virus‐neutralizing antibody activity and demonstrate the therapeutic potential of the Fc‐free strategy that can be used advantageously to provide both immediate and long‐term protection against SARS‐CoV‐2 and other viral infections.

## Graphical abstract

Here they generate a bispecific Fc‐free trimeric antibody to neutralize and target SARS‐CoV‐2 virions to type 1 conventional dendritic cells and promote T cell cross‐priming. Therapeutic administration protected mice from a lethal infection, boosting virus‐specific humoral and CD8^+^ T cell immune responses. The results highlight the potential of bispecific trimerbodies with neutralizing and vaccine‐like action to provide both immediate and long‐term protection against SARS‐CoV‐2 and other viral infections.

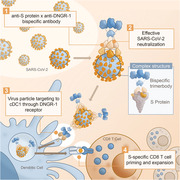

## 1 Introduction

Coronavirus disease 2019 (COVID‐19), caused by severe acute respiratory syndrome coronavirus 2 (SARS‐CoV‐2), has given rise to one of the worst pandemics in recent history. As July 2023, the virus has infected more than 767 million individuals, causing over 6.9 million deaths (https://covid19.who.int/). Although multiple effective vaccines preventing COVID‐19 are being widely administered worldwide,^[^ ^1^, ^2^, ^3^ ^]^ the emergence of multiple SARS‐CoV‐2 variants causing increased viral dispersion and immune evasion requires the continual development of effective therapeutics against COVID‐19.^[^ ^4^, ^5^, ^6^ ^]^ In this context, monoclonal antibodies (mAb) have shown efficacy in animal models of SARS‐CoV‐2 infection^[^ ^7^, ^8^, ^9^ ^]^ and several mAb‐based therapeutics received Emergency Use Authorization.^[^ ^10^, ^11^, ^12^ ^]^

SARS‐CoV‐2 infection requires the spike (S) protein receptor‐binding domain (RBD) docking to the cell surface receptor angiotensin‐converting enzyme 2 (ACE2) for viral entry into host cells,^[^ ^13^, ^14^, ^15^ ^]^ so most SARS‐CoV‐2‐neutralizing antibodies (nAb) block this interaction by direct binding to the RBD.**^[^** ^16^ **^,^** ^17^ **^]^** Accordingly, viral strains with mutations altering the RBD surface can avoid antibody recognition and neutralization. A particularly negative effect has been attributed to the K417N/T, L452R, T478K, and E484K/Q mutations, as seen in the B.1.351 (beta), P.1 (gamma), B.1.617.2 (delta), or B.1.1.529 (omicron) variants of concern (VOCs). The spread of these VOCs has reduced the efficacy of vaccines and mAb‐based therapeutics,^[^ ^4^, ^18^, ^19^ ^]^ making it mandatory to update them against present and future SARS‐CoV‐2 variants.

Camelid‐derived single‐domain antibodies, also known as V_HH_s or nanobodies, combine antigen‐binding affinities that are comparable to conventional antibodies with a smaller size (15 kDa), high stability, and engineering simplicity.^[^ ^20^, ^21^ ^]^ Their potential for use against SARS‐CoV‐2 infection has been widely reported both in vitro^[^ ^22^, ^23^, ^24^, ^25^ ^]^ and in vivo.^[^ ^26^, ^27^, ^28^ ^]^ Many multimerization strategies have been used to increase their potency, such as bispecific and biparatopic fusions,^[^ ^23^, ^29^, ^30^ ^]^ V_HH_‐Fc constructs^[^ ^22^, ^24^ ^]^ and N‐terminal V_HH_‐based trimerbodies.^[^ ^31^ ^]^ Some of these multimerized V_HH_s completely neutralize the infectivity of SARS‐CoV‐2 and even suppress the emergence of escape mutants.^[^ ^23^, ^29^, ^31^ ^]^

Dendritic cells (DCs) are professional antigen‐presenting cells that play a central role in the induction of antigen‐specific adaptive immune responses during infection.^[^ ^32^ ^]^ C‐type lectin receptors (CLR), such as DEC‐205, DCIR‐2 and DC‐SIGN, have been increasingly used in preclinical models for in vivo targeting of antigens to DCs.^[^ ^33^, ^34^, ^35^, ^36^ ^]^ Dendritic cell natural killer lectin group receptor‐1 (DNGR‐1), encoded by the gene *Clec9a*, is a CLR selectively expressed at high levels by mouse CD8α^+[^ ^37^ ^]^ and CD103^+^ DCs,^[^ ^38^ ^]^ and by their human equivalents.^[^ ^39^ ^]^ In this DC subset, defined as conventional type 1 DCs (cDC1s), DNGR‐1 promotes cross‐priming of cytotoxic CD8*^+^* T cell (CTL) responses by diverting of necrotic cell cargo into a recycling endosomal compartment, preferentially resulting in major histocompatibility complex class I cross‐presentation to CTLs.^[^ ^40^, ^41^ ^]^ Therefore, DNGR‐1 may be used as a target to enhance anti‐viral CTL responses by specific‐priming of cDC1s with viral components, such as antigens or whole virions.^[^ ^42^ ^]^

Here, we report the development of TN^T^, a SARS‐CoV‐2‐nAb comprising an anti‐RBD biparatopic tandem‐nanobody (TN) integrated in a trimerbody scaffold.^[^ ^43^ ^]^ It neutralized Wuhan‐Hu‐1/B.1 lineage S protein‐pseudotyped vesicular stomatitis virus (VSV) and live SARS‐CoV‐2 viruses 10‐ and 20‐fold more effectively, respectively, than the monomeric TN and demonstrated potent neutralization of the K417N/T, E484K, N501Y, and L452R antibody‐escape mutations found in the beta, gamma, and delta VOCs. In a second engineering step, the DNGR‐1‐specific 7H11 single‐chain variable fragment (scFv) was C‐terminally fused to TN^T^ to target SARS‐CoV‐2 neutralized virions to DCs and induce virus‐specific CTL responses. This bispecific TN^T^DNGR‐1 antibody selectively targeted viral antigens to DCs expressing DNGR‐1, promoting their receptor‐mediated internalization. In a prime‐boost regime coadministration in mice of TN^T^DNGR‐1 and S protein significantly improved antiviral S‐specific responses, enhancing the generation of SARS‐CoV‐2 S‐specific CD8^+^ T cells and the polarization of the humoral responses towards the pro‐Th1 response IgG2c subclass. Remarkably, intraperitoneal administration of TN^T^DNGR‐1, but not of TN^T^, in SARS‐CoV‐2 infected K18‐hACE2 mice protected all mice from lethal SARS‐CoV‐2 challenge. TN^T^DNGR‐1 reduced viral load in the lungs, increased IgG and IgM antibody titers against S protein and other viral antigens in serum samples and mediated effective SARS‐CoV‐2 neutralizing activity. Particularly, TN^T^DNGR‐1 treatment boosted the generation of S‐specific CD8^+^ T cells in the lungs, indicating an enhancement of T cell‐mediated antiviral responses.

## 2 Results

### 2.1 Design of a Broadly Neutralizing Biparatopic Trimeric Antibody Targeting the SARS‐CoV‐2 S Protein RBD

**Figure 1:**
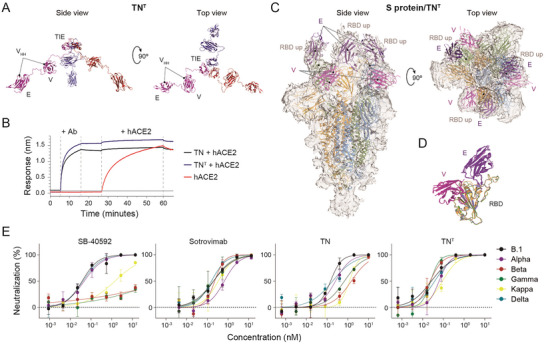
The TN^T^ trimerbody efficiently blocks RBD‐hACE2 interaction by simultaneous binding to the three RBDs of the SARS‐CoV‐2 S protein causing broad effective neutralization activity. A) Hypothetical model of the TN^T^ trimerbody displayed in lateral and top views showing V_HH_s and TIE trimerization domain disposition. Each monomer was colored differently (blue, red and purple). B) The ability of TN and TN^T^ antibodies to block the interaction of ACE2 with RBD was investigated using BLI. TN (black) or TN^T^ (blue) were associated with immobilized B.1 RBD, after which ACE2 was added. Unblocked control is also shown (red). C) Side and top views of sharpened cryo‐EM map corresponding to the trimeric S protein ectodomain after incubation with TN^T^. The map is shown in pale grey and the S protein/TN^T^ modelization is included in the map as cartoon representation. Each protomer of the S protein (3‐up RBD) is colored differently (yellow, blue, and green) and the docked domains, E and V V_HH_s forming the three‐arms of the TN^T^, bound simultaneously to the three RBDs are shown in purple and magenta, respectively. D) The alignment of the three RBDs bound to E and V V_HH_s from our model illustrates the similar way of interaction between the three arms of TN^T^ and each RBD. E) Neutralization of SARS‐CoV‐2 S‐pseudotyped rVSV‐luc. Twofold serial dilutions of control nAb (SB‐40592 or sotrovimab), TN or TN^T^ were incubated with pseudoviruses expressing S protein from different variants prior to Vero E6 cells infection. Normalized values from three independent experiments ± SEM are plotted. Overall, there was an excellent correlation between the three neutralization assays (*R* ^2^ = 0.92).

For the generation of the biparatopic V_HH_‐tandem neutralizing antibody (TN), we used two well‐characterized neutralizing V_HH_s (E and V) that recognize two non‐overlapping epitopes on the SARS‐CoV‐2 Wuhan‐Hu‐1/B.1 RBD as building blocks,^[^ ^23^ ^]^ fusing them with a 15‐amino acid‐long (G_4_S)_3_ linker. To improve TN's binding to RBD through multivalency (i.e., the avidity effect) and to increase RBD occupancy within the S protein trimer, TN was integrated into a trimerbody scaffold by fusing it to a human collagen XVIII‐derived homo‐trimerization (TIE) domain^[^ ^44^ ^]^ generating a biparatopic V_HH_‐based trimerbody (TN^T^) (**Figure** **1**A). TN and TN^T^ were purified from HEK‐293 conditioned media, showing migration patterns in SDS‐PAGE under reducing conditions consistent with their theoretical molecular weights (29.6 and 38 kDa, respectively) (Figure S1A, Supporting Information). In a size exclusion chromatography‐multiangle light scattering (SEC‐MALS) analysis TN eluted as a major peak with a molar mass of 31 kDa and TN^T^ as a single peak with 118 kDa (calculated masses are 29.6 and 114 kDa, respectively) (Figure S1B, Supporting Information). Circular dichroism (CD) spectra (Figure S1C, Supporting Information) display minima at 217 nm, characteristic of β‐sheet secondary structure. The cooperative thermal denaturation curves, with mid‐point temperatures >60 °C (Figure S1D, Supporting Information), indicate stable three‐dimensional structures in both antibodies.

### 2.2 TN‐Based Antibodies Bind SARS‐CoV‐2 S Protein RBD with High Affinity and Efficiently Block the RBD‐ACE2 Interaction

Biolayer interferometry (BLI) analysis confirmed the ability of TN and TN^T^ to bind B.1 RBD and block its interaction with human ACE2 (hACE2). Treating the biosensor‐immobilized B.1 RBD with any of the two antibodies prevented nearly all (>95%) subsequent binding of hACE2 (Figure 1B). Moreover, TN and TN^T^ exhibited similar reactivity against B.1.351 (beta), P.1 (gamma), B.1.617.1 (kappa), and B.1.617.2 (delta) RBDs compared to the B.1 RBD, as measured by enzyme‐linked immunosorbent assay (ELISA) (Figure S1E, Supporting Information). BLI analysis confirmed that TN and TN^T^ bind with high affinity to immobilized B.1 RBD (Figure S1F, Supporting Information) and demonstrated negligible dissociation over one hour of measurement. This precluded a precise determination of their dissociation rate but was indicative of high‐affinity sub‐nanomolar dissociation constants (*K*_D_). As expected from their respective monomeric and trimeric natures, TN^T^ association rate constant was approximately threefold greater than for TN (*k*_a_ values of ≈16 and ≈6 × 10^4^ M^−1^ s^−1^, respectively). A similar study was performed where both TN and TN^T^ bound the beta variant RBD essentially identically to the B.1 RBD (Figure S1G, Supporting Information).

### 2.3 Biparatopic TN^T^ Binds Simultaneously to all Three S Protein RBDs

The stoichiometry of the interaction between S protein and TN^T^ was studied by microfluidic diffusional sizing (MDS) analysis. The hydrodynamic radius (*R*_h_) of TN^T^ (5.06 nm) is consistent with its trimeric form, increasing to 16.6 nm when mixed with equimolar amount of HexaPro S protein. This indicates that TN^T^ binds to trimeric S protein at a 1:1 stoichiometry (Figure S2A,B, Supporting Information). Cryo‐electron microscopy (cryo‐EM) was used to determine the structure of the HexaPro S protein/TN^T^ complex (Figure S2C–H; see Experimental Section in the Supporting Information for details). An electron density map with 3.8 Å resolution was used to build a consistent model of the S protein ectodomain (yellow, blue and green, Figure 1C). While the isolated S protein exists predominantly in a closed RBD conformation,^[^ ^14^ ^]^ the TN^T^‐bound S protein is mainly in the prefusion state with all three RBDs in the open conformation (Figure 1C; Figure S3A,B, Supporting Information). The TIE domain and the linkers of TN^T^ were not observed (likely due to their flexibility and lack of direct binding to the S protein), but there were densities in the map that made it possible to model the six TN^T^ V_HH_s bound to their RBD epitopes (Figure 1C,D; Figure S3A,B, Supporting Information). As the length of the 15‐amino acid linker connecting the C‐terminus of E V_HH_ and the N‐terminus of V V_HH_ (53 Å in an extended conformation) is compatible with this distance between the E and V V_HH_s binding to the same RBD (43 Å), but not compatible with those bound to different RBDs (>90 Å), each tandem V_HH_ must interact with a single RBD. In concert, the three tandem V_HH_s of TN^T^ completely block all of the S protein's sites of interaction with the hACE2 receptor. The RBD binding by the E and V V_HH_s within the TN^T^ is very similar to that of the monomeric tandem V_HH_s, which was previously described (PDB: 7B18),^[^ ^23^ ^]^ although in the S protein/TN^T^ model there is a major shift in the position of one of the RBDs relative to the other two in the complex (Figure S3C, Supporting Information).

### 2.4 Trimerization Enhances SARS‐CoV‐2 Neutralization by the TN Antibody and Prevents Escape Caused by Mutations Found in Most VOCs

The neutralizing activity of TN and TN^T^ was assessed using replication‐deficient G‐luciferase VSV pseudotyped with the SARS‐CoV‐2 S protein. SB‐40592 and sotrovimab biosimilar were used as control mAbs. SB‐40592 neutralized S protein‐pseudotyped particles with half‐maximal effective concentration (EC_50_) of 37 × 10^−12^ m for B.1, 47 × 10^−12^ m for VOC B.1.1.7 (alpha) and 41 × 10^−12^ m for VOC delta, but was ineffective against beta, gamma, and kappa strains (Figure 1E). Sotrovimab EC_50_ values were between 156 and 335 × 10^−12^ m for all assayed strains (Figure 1E). TN neutralized infection strongly in a dose‐dependent manner, with EC_50_ values of 147 and 182 × 10^−12^ m for the B.1 and delta pseudoviruses, respectively; however, its efficacy was significantly decreased against beta (approximately tenfold), kappa (approximately eightfold), and gamma (approximately fivefold) variants (Figure 1E). The trimerization‐conveyed avidity of TN^T^ reduced the EC_50_ concentration against B.1 pseudovirus to 26 × 10^−12^ m (approximately sixfold) and it retained this efficacy against all the variants studied. Specifically, TN^T^ was nearly 70‐ and 30‐fold more effective than TN against beta and gamma VOCs (EC_50_ of 23 and 24 × 10^−12^ m, respectively) (Figure 1E).

### 2.5 Design of a SARS‐CoV‐2‐Neutralizing Trimerbody Targeting Dendritic Cells

**Figure 2:**
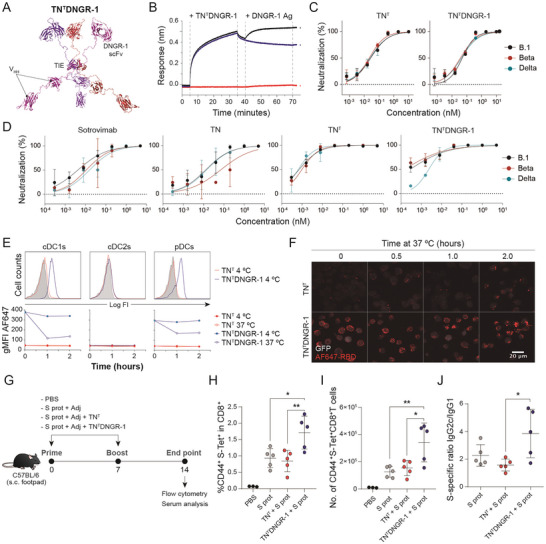
The bispecific TN^T^DNGR‐1 trimerbody combines potent and broad SARS‐CoV‐2 neutralization with specific targeting of viral antigens to DNGR‐1‐expressing DCs for boosting systemic S‐specific effector CD8^+^ T cell responses. A) TN^T^DNGR‐1 hypothetic model showing V_HH_‐tandem, TIE trimerization domain and anti‐DNGR‐1 7H11 scFv antibody. Each polypeptide chain is represented in one different color (blue, red and purple). B) Bispecificity of TN^T^DNGR‐1 was investigated using BLI by incubating RBD‐coated biosensors first with antibody, and then with DNGR‐1. Control RBD‐coated biosensors with TN^T^DNGR‐1 but without DNGR‐1 and just with DNGR‐1 were also included. C) Neutralization of SARS‐CoV‐2 S‐pseudotyped rVSV‐luc. Twofold serial dilutions of each trimerbody were incubated with SARS‐CoV‐2 S‐pseudotyped rVSV‐luc expressing S protein from beta and delta variants prior to infecting Vero E6 cells. D) B.1, beta and delta SARS‐CoV‐2 virus neutralization of infection. Five‐fold serial dilutions of sotrovimab, TN, TN^T^ or TN^T^DNGR‐1 were incubated with live viruses prior to infecting Vero E6/TMPRSS2 cells. E) Flow cytometry analysis of Flt3‐L BMDCs after incubation at 4 or 37 °C with TN^T^DNGR‐1 or control TN^T^ and subsequent stained with AF647‐RBD staining and antibodies to discriminate DC subsets. Upper histograms represent actual staining for the different cell subsets at 1 h and 4 °C. Lower data represent geometric mean fluorescence intensity (gMFI) for the cDC1, cDC2, and pDC subsets at the different conditions. F) Representative pictures of MuTu‐DC cells incubated with TN^T^DNGR‐1 or control TN^T^ and AF647‐RBD at 4 °C and then for different times at 37 °C before fixation and visualization using confocal microscopy. G) Immunization of C57BL/6 mice using a prime‐boost scheme with either: (1) PBS; (2) S protein + adjuvants (poly I:C + CpG); (3) S protein + TN^T^ + adjuvants; or (4) S protein + TN^T^DNGR‐1 + adjuvants. One representative from two independent experiments (*n* = 3–5 mice/group /experiment) is shown. Spleens were harvested on day 14 and stained for S‐specific CD8^+^ T cells using specific S protein tetramer, H‐2Kb (539‐VNFNFNGL‐546) (S‐Tet). H) Frequency of CD44^+^ S‐Tet^+^ cells within the T CD8 gate and I) total number of CD44^+^ S‐Tet^+^ CD8^+^ T cells are shown. *p < 0.05; **p < 0.01 by one‐way ANOVA followed by Tukey's multiple comparison test. CD44^+^ S‐Tet^+^ representative dot plots gated on CD8^+^ T cells for the different treatment groups are presented in Figure S4F in the Supporting Information, G,J) The S‐specific IgG2c/IgG1 ratio by mean of serum S‐specific subclass IgG determination by ELISA at a 1/250 dilution in serum samples obtained on day 14. Raw data for IgG1 and IgG2c, in serum samples diluted 1/250 are presented in Figure S4H in the Supporting Information. *p < 0.05 by one‐way ANOVA followed by Tukey's multiple comparison test.

To allow TN^T^ to prime adaptive immune responses in addition to its short‐term virus‐neutralizing capacity, we aimed to deliver the TN^T^‐bound virus to cDC1s, which excel at priming anti‐viral responses. With this purpose, we generated a bispecific trimerbody (TN^T^DNGR‐1) by fusing the anti‐DNGR‐1 7H11 scFv^[^ ^37^ ^]^ to the C‐terminus of TN^T^ through a flexible 15‐amino acid linker (**Figure** **2**A). SDS‐PAGE analysis of purified TN^T^DNGR‐1 revealed a single band consistent with its estimated molecular weight (64.7 kDa) (Figure S4A, Supporting Information) and SEC‐MALS analysis showed one major symmetric peak with a molar mass of 175 kDa (Figure S4B, Supporting Information), close to its theoretical trimeric mass of 194 kDa. BLI studies proved that TN^T^DNGR‐1 bound to immobilized B.1 RBD with high affinity (Figure 2B) and could bind soluble DNGR‐1 while remaining bound to the immobilized RBD, demonstrating its ability to bind both antigens simultaneously (Figure 2B). TN^T^DNGR‐1 bound B.1 RBD in ELISA assays and HEK‐293 cells expressing B.1 S protein as efficiently as TN^T^ (Figure S4C,D, Supporting Information). Furthermore, in accordance with its high‐affinity binding to immobilized RBD protein, TN^T^DNGR‐1 neutralized pseudovirus infection as efficiently as TN^T^, with EC_50_ values of 49.2, 36.9, and 42.6 × 10^−12^ m for B.1, beta and delta variants, respectively (Figure 2C). Moreover, TN inhibited SARS‐CoV‐2 infection of Vero E6/TMPRSS2 cells in a similar way to sotrovimab for B.1 and delta strains, but its EC_50_ was 5‐fold increased against beta VOC. Comparatively, TN^T^DNGR‐1 and TN^T^ promoted a higher neutralization effect than monomeric TN and sotrovimab for all assayed variants (Figure 2D). Both trimerbodies avoid viral escape with EC_50_ of 0.3 and 0.7 × 10^−12^ m for B.1 strain, 0.15 and 1 × 10^−12^ m for VOC beta, and 2.3 and 0.64 × 10^−12^ m for VOC delta, respectively (Figure 2D). Comparatively sotrovimab EC_50_ values for these strains were 21, 11, and 16 × 10^−9^ m, respectively (Figure 2D).

### 2.6 TN^T^DNGR‐1 Trimerbody Targets RBD to DNGR‐1‐Expressing Dendritic Cells Promoting its Internalization

To test the specificity of TN^T^DNGR‐1 we stained B3Z and B3Z^DNGR‐1^ cells with AF647‐conjugated B.1 RBD (AF647‐RBD) preincubated with TN^T^ or TN^T^DNGR‐1 and found that B3Z^DNGR‐1^ cells were labeled by AF647‐RBD only in the presence of TN^T^DNGR‐1 but not the TN^T^ control (Figure S4E, Supporting Information). To address whether TN^T^DNGR‐1 (as well as neutralized RBD or virions) is internalized by DCs, we incubated Flt3L bone marrow‐derived dendritic cells (BMDCs) with TN^T^ or TN^T^DNGR‐1 for up to two hours at 4 or 37 °C and then added AF647‐RBD for detecting cell surface‐bound trimerbodies. DNGR1‐expressing DCs, mainly cDC1s but also plasmacytoid (pDCs), were stained with AF647‐RBD when DCs were preincubated with TN^T^DNGR‐1, but not with TN^T^, at 4 °C (Figure 2E; Figure S4F, Supporting Information). Notably, after 1‐ or 2‐h incubation with TN^T^DNGR‐1 at 37 °C, the AF647‐RBD signal was reduced 4‐fold in these subpopulations, suggesting its internalization (Figure 2E, lower graphs). The ability of TN^T^DNGR‐1 to promote cell internalization of soluble RBD was confirmed in MuTu‐DCs (a mouse DC cell line expressing DNGR‐1) by confocal microscopy. Only cell preparations treated with TN^T^DNGR‐1, but not with TN^T^, followed by immediate AF647‐RBD incubation showed specific staining and internalization of the TN^T^DNGR‐1/AF647‐RBD complex after 2 h of incubation at 4 or 37 °C (Figure 2F).

### 2.7 TN^T^DNGR‐1 Trimerbody Increases S Protein‐Specific CD8^+^ T Cells in Immunocompetent Mice

We investigated whether TN^T^DNGR‐1 could increase the generation of adaptive immunity by specific targeting of captured SARS‐CoV‐2 S protein towards cDC1s. We treated immunocompetent C57BL/6 mice in a prime‐boost scheme with the following stimuli: 1) PBS; 2) S protein + adjuvants (poly I:C + CpG); 3) S protein + TN^T^ + adjuvants; or 4) S protein + TN^T^DNGR‐1 + adjuvants (Figure 2G). Immunization with S protein, like S protein + TN^T^, led to a significant increase in the generation of systemic S protein‐specific effector CD8^+^ T cells quantified by S protein‐specific tetramer (S‐Tet) staining, compared to PBS‐treated control mice (Figure 2H,I; Figure S4G, Supporting Information). Notably, immunization with S protein in the presence of TN^T^DNGR‐1 resulted in a further increase of the frequency and number of S protein‐specific effector CD8^+^ T cells (Figure 2H,I; Figure S4G, Supporting Information). Moreover, ELISA analysis in serum samples reveals that all the S protein treated mice groups induced potent S protein‐specific IgM and total IgG antibody responses compared to PBS‐treated mice (Figure S4H, Supporting Information). By contrast, RBD‐specific IgM and total IgG antibody responses were only induced in mice immunized with S protein and with S protein + TN^T^DNGR‐1, but not in animals immunized with S protein + TN^T^ or PBS‐treated mice (Figure S4I, Supporting Information). Interestingly, the measurement of S protein‐specific IgG1 and IgG2c responses (Figure S4H, Supporting Information) showed that S protein + TN^T^DNGR‐1 administration induced lower levels of S protein‐specific IgG1, increasing the ratio of IgG2c to IgG1, suggesting a fostered Th1‐polarized antibody profile response (Figure 2J). RBD‐specific IgG1 and IgG2c responses were only enhanced compared PBS by the immunization with S protein, but no significant differences were found for TN^T^ and TN^T^DNGR‐1 coadministration groups (Figure S4I, Supporting Information).

### 2.8 TN^T^DNGR‐1 Trimerbody Protects Against a Lethal SARS‐CoV‐2 Challenge

**Figure 3:**
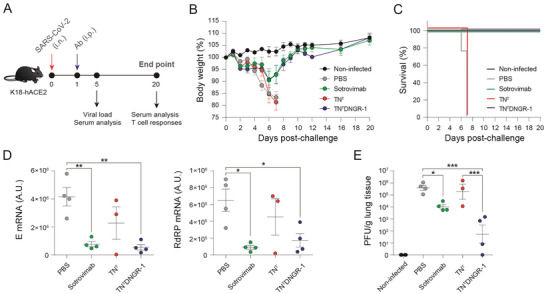
Therapeutic application of TN^T^DNGR‐1, but not TN^T^, protects mice of SARS‐CoV‐2 lethal infection by reducing viral load in the lungs. A) Efficacy experiment in K18‐hACE2 mice was done using a single antibody (Ab) intraperitoneal (i.p.) administration 17 h after intranasal (i.n.) viral challenge (10^5^ PFUs/mouse). Experimental groups (*n* = 8 mice/group, 1 experiment) included: uninfected control group (black) and infected groups treated with PBS (grey), sotrovimab biosimilar (green), TN^T^ (red), and TN^T^DNGR‐1(blue). On day 5 p.c. 4 mice per group were euthanized to obtained serum samples and lungs (*n* = 3 in the case of the TN^T^ group). Serum was collected on day 5 and 20 post‐challenge (p.c.) for humoral immune response analysis. Lungs were harvested on day 5 p.c. for viral load determination and on day 20 p.c. for the analysis of S‐specific T cell responses. B) Weight change after B.1 (isolate MAD6) SARS‐CoV‐2 challenge followed by antibody administration (mean ± SEM) expressed as percentage of initial body weight. C) Kaplan‐Meyer survival representation of the efficacy experiment. D) Viral mRNA levels at day 5 p.c. in the right lung measured for E (envelope) and RNA‐dependent RNA polymerase (RdRP) genes. E) Infective virus in the lungs at day 5 p.c. represented as PFUs/g of lung tissue. All graphs represent individual values plus mean ± SEM for a single experiment. *p < 0.05; **p < 0.01; ***p < 0.001 by one‐way ANOVA followed by Tukey's multiple comparison test.

To evaluate the capacity of the TN^T^DNGR‐1 trimerbody to protect against SARS‐CoV‐2 infection, we performed an in vivo protection study in K18‐hACE2 transgenic mice, which express the human ACE2, and are therefore susceptible to SARS‐CoV‐2 infection (**Figure** **3**).^[^ ^45^ ^]^ K18‐hACE2 mice (*n* =  8/group) were challenged intranasally (i.n.) with a lethal dose (1 × 10^5^ plaque‐forming units (PFUs)/mouse) of SARS‐CoV‐2 (isolate MAD6, a prototypic B.1 strain) and 17 h later were treated by intraperitoneal (i.p.) injection of TN^T^, TN^T^DNGR‐1, sotrovimab or PBS (as a nontreated infected control). In addition, noninfected and nontreated mice were used as a control group of healthy animals (Figure 3A). Mice were monitored for 20 d to determine body weight loss and survival (Figure 3B,C). All treated animals lost approximately 5% to 8% of their initial body weight during the first days postchallenge (p.c.), 10% to 13% in the case of the control PBS‐treated group, but by day 6 p.c. mice treated with TN^T^DNGR‐1 or sotrovimab began to recover body weight reaching the baseline body weight by day 9 p.c. (Figure 3B) and all survived (Figure 3C). However, PBS‐ and TN^T^‐treated mice continue to lose body weight (Figure 3B) and succumbed or were euthanized on day 7 p.c. (Figure 3C). Next, to assess the impact of TN^T^DNGR‐1 treatment on the replication of SARS‐CoV‐2, four mice per group were sacrificed on day 5 p.c., the lungs were harvested and processed, and the presence of SARS‐CoV‐2 subgenomic E and genomic RdRp RNA (Figure 3D), and infectious virus (Figure 3E) analyzed. Treatment with TN^T^DNGR‐1 and sotrovimab significantly reduced the subgenomic and genomic SARS‐CoV‐2 RNA levels in the lungs compared to PBS‐ or TN^T^‐treated mice (Figure 3D). Live infectious virus determination showed that TN^T^DNGR‐1 induced a more potent reduction than sotrovimab in virus titers in the lungs, whereas PBS‐ and TN^T^‐treated mice show similar titers (Figure 3E).

### 2.9 TN^T^DNGR‐1 Trimerbody Induces SARS‐CoV‐2‐Specific Humoral and Cellular Immune Responses

**Figure 4:**
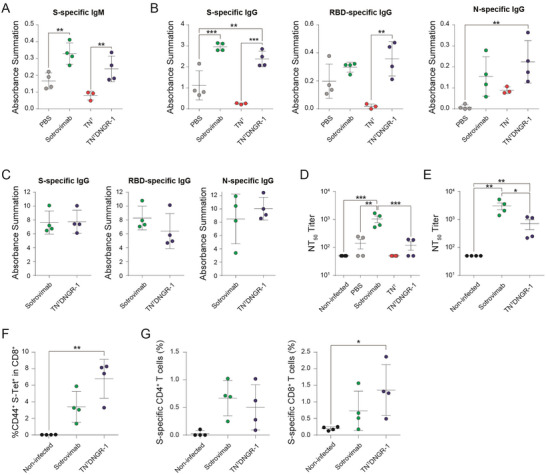
TN^T^NDGR‐1 mediates an effective humoral and cellular immune response against SARS‐CoV‐2, specifically by providing enhanced S‐specific cytotoxic T CD8^+^ cell responses in the lungs. A) Anti‐S IgM levels in serum 5 d p.c. expressed as absorbance summation. B) Quantification of S‐, RBD‐, and N‐specific total IgG levels at 5 d p.c. expressed as absorbance summation. C) Quantification of S‐, RBD‐, and N‐specific total IgG levels at 20 d p.c. expressed as absorbance summation. D,E) SARS‐CoV‐2 neutralization. Serum neutralization of live SARS‐CoV‐2 (B.1 strain), expressed as NT_50_, at day 5 (D) and 20 (E) p.c. Lungs were harvested on day 20 p.c. and stained for S‐specific CD8^+^ T cells using specific S protein tetramer H‐2Kb (539‐VNFNFNGL‐546) (S‐Tet). F) Frequency of CD44^+^ S‐Tet^+^ cells within the T CD8 gate. G) Frequency of S‐specific T CD4^+^ (left panel) and T CD8^+^ cells (right panel) in the lungs at 20 d p.c. and evaluated by an intracellular cytokine staining assay measuring expression of CD107a, and secretion of IFN‐γ, TNF‐α, and IL‐2. All graphs represent individual values plus mean ± SEM (*n* = 4 mice/group; *n* = 3 mice in the case of TN^T^ at day 5 p.c.) for a single experiment. *p < 0.05; **p < 0.01; ***p < 0.001 by one‐way ANOVA followed by Tukey's multiple comparison test. *p < 0.05 for S‐specific CD8^+^ T cells analysis (G) was done by Dunn's multiple comparisons test following Friedman test.

Next, to study whether TN^T^DNGR‐1 could promote SARS‐CoV‐2‐specific adaptive immunity, we analyzed the humoral and cellular immune responses induced in K18‐hACE2 mice. On day 5 p.c., mice treated with TN^T^DNGR‐1 and sotrovimab induced significantly higher titers of S‐specific IgM (**Figure** **4**A; Figure S5A, Supporting Information) and S‐, RBD‐, and N‐specific IgG antibodies than nontreated or TN^T^‐treated mice (Figure 4B; Figure S5B–D, Supporting Information). Furthermore, on day 20 p.c., mice treated with TN^T^DNGR‐1 or sotrovimab both elicited similar high titers of S‐, RBD‐, and N‐specific IgG antibodies (Figure 4C; Figure S5E–G, Supporting Information), which were highly increased when compared to the levels induced on day 5 p.c. Treatment with TN^T^DNGR‐1 and sotrovimab led to higher titers of serum nAb against live SARS‐CoV‐2 on day 5 p.c, than PBS‐ or TN^T^‐treated mice. (Figure 4D; Figure S6A, Supporting Information). Consistently and similar to IgG antibody titers at day 20 p.c., neutralizing antibody titers increased highly in TN^T^DNGR‐1‐ and sotrovimab‐treated mice compared to day 5 p.c. levels (Figure 4E; Figure S6B, Supporting Information). To assess the contribution of the administrated antibodies to this neutralizing activity, we determined the concentration of TN^T^, TN^T^DNGR‐1 and sotrovimab in mice serum at day 5 and 20 p.c., obtaining significant higher levels for sotrovimab (8.26 µg mL^−1^ and 12.72 ng mL^−1^, respectively) than for TN^T^DNGR‐1 (38.73 and 0.11 ng mL^−1^, respectively) and TN^T^ (0.32 ng mL^−1^ and undetermined, respectively) (Figure S6C, Supporting Information).

The study of T cell responses by S‐Tet staining in the lungs obtained on day 20 p.c. showed that TN^T^DNGR‐1 treatment selectively induced a significant local increase of S protein‐specific effector CD8^+^ T cells compared to non‐infected mice (Figure 4F). Notably, the magnitude of this effect was superior to that promoted by sotrovimab in the same non‐infected mice (Figure 4F). In addition, the presence of S‐specific CD4^+^ and CD8^+^ T cell immune responses was further assessed by an intracellular cytokine staining (ICS) assay in lungs cells obtained on day 20 p.c. and restimulated in vitro with SARS‐CoV‐2 S peptide pools. Treatment with TN^T^DNGR‐1 and sotrovimab induced S‐specific CD4^+^ and CD8^+^ T cells expressing the cytotoxic marker CD107a and/or secreting IFNγ, TNFα, and/or IL‐2 cytokines (Figure 4G). While TN^T^DNGR‐1 and sotrovimab elicited similar levels of S‐specific CD4^+^ T cells, TN^T^DNGR‐1‐treatment specifically triggered a significantly increased number of S‐specific CD8^+^ T cells compared to non‐treated group (Figure 4G). In detail, TN^T^DNGR‐1 triggered a trend towards a higher amount of S‐specific CD8^+^ T cells individually secreting IFNγ, TNFα, or expressing CD107a compared to sotrovimab, but did not show any differences in the CD4^+^ T cell subset (Figure S7E, Supporting Information). Together, these results suggest that the addition of DNGR‐1 specificity to the TN^T^ moiety redirects the virus to cDC1s and favors cross‐priming and a boosted adaptive response against the S protein of SARS‐CoV‐2.

## 3 Discussion

To date, most mAb‐based therapeutic strategies against SARS‐CoV‐2 have focused on virus neutralization and clearance, either with conventional mAbs or by implementing multispecificity through Fc fusion or other multimerization strategies.^[^ ^10^, ^29^, ^46^, ^47^, ^48^ ^]^ Furthermore, it has been widely demonstrated in SARS‐CoV‐2 infection in vivo models that virus‐neutralizing antibody activity depends on Fc‐FcγR interactions.^[^ ^49^, ^50^, ^51^ ^]^ Different approaches of nAbs with Fc‐optimized binding to FcγIIa and FcγIIIa receptors have been described with superior potency to prevent or treat COVID‐19 disease.^[^ ^10^, ^52^ ^]^ Nonetheless, the lately emergence of SARS‐CoV‐2 strains, showing reduced sensitivity to both vaccine‐elicited and clinically administrated nAbs, or the upcoming emergence of other viruses with pandemic potential demands the development of innovative approaches for the effective treatment of viral infections.

In this study, we evaluated the potential of an Fc‐free bispecific antibody, TN^T^DNGR‐1, incorporating an anti‐RBD V_HH_‐tandem (TN) and an anti‐DNGR‐1 scFv into a trimerbody scaffold, for priming virus‐specific CD8^+^ T cell responses and protect transgenic K18‐hACE2 mice against SARS‐CoV‐2 infection. Although vaccine‐like approaches targeting specific tumor or viral antigens to DCs by fusing them to mAbs have been previously described,^[^ ^37^, ^53^ ^]^ a strategy that combines immediate virus neutralization with specific cDC1s targeting, aiming to accelerate and boost adaptive immune responses during infection, has never been explored.

RBD‐targeted V_HH_s have shown effective in neutralizing SARS‐CoV‐2 infection in vitro and in protecting animals from SARS‐CoV‐2 challenge,^[^ ^23^, ^25^, ^27^, ^28^, ^54^ ^]^ making them a valuable alternative for the development of multivalent and multiparatopic agents (targeting multiple viral epitopes) with improved neutralization efficiency and decreased vulnerability to escape mutations.^[^ ^29^, ^55^, ^56^ ^]^ Here, we used the high‐affinity biparatopic V_HH_‐tandem, TN, previously described to potently neutralize SARS‐CoV‐2 strain Wuhan‐Hu‐1 and prevent the emergence of escape mutants.^[^ ^23^ ^]^ To maximize its neutralizing efficacy instead of generating a conventional Ig‐like Fc‐fusion,^[^ ^24^, ^26^, ^56^ ^]^ we generated an anti‐RBD hexavalent nAb, TN^T^, made by fusing TN to the human collagen XVIII homo‐trimerization domain, an effective scaffold for the generation of trimeric antibodies for clinical use.^[^ ^44^ ^]^

TN^T^ demonstrated a complete, potent blocking of the RBD‐hACE2‐ interaction and a strong similar binding to beta, gamma, kappa, and delta variants RBDs. Furthermore, compared to TN, TN^T^ revealed a significant improvement in neutralization efficacy against all tested VOCs (six‐ and fivefold for B.1 and delta, respectively), but especially for those containing E484K/Q mutation, as beta and gamma (70‐ and 30‐fold superior, respectively). Compared to sotrovimab,^[^ ^10^, ^57^ ^]^ the only formerly clinically used nAb active against all VOCs, TN^T^ shows three‐ to tenfold induction in neutralization potency against all studied VSV‐pseudotyped strains and TN^T^DNGR‐1 efficacy was almost identical to the parental TN^T^ antibody (B.1, 49 vs 26 × 10^−12^ m; beta, 36 vs 22 × 10^−12^ m, and delta, 42 vs 38 × 10^−12^ m, respectively). Similar results were obtained in live‐virus neutralization, where TN^T^DNGR‐1 and TN^T^ trimerbodies exhibited a more potent neutralization activity than TN and sotrovimab against B.1, beta and delta VOCs, with 10‐ to 100‐fold and 6‐ to 50‐fold reductions in EC_50_ values. Together with the structural data for the S protein:TN^T^ complex, showing an equimolar (1:1) interaction mechanism between TN^T^ and the trimeric S protein, where both V_HH_s of each TN arm bind simultaneously to the 3‐RBDs, our functional results demonstrate that TN^T^ scaffold represent an optimal design for the neutralization of viruses presenting trimeric proteins for receptor attachment, such as SARS‐CoV‐2, influenza, respiratory syncytial virus and human immunodeficiency 1 virus.^[^ ^58^, ^59^, ^60^ ^]^ Thereby, although TN^T^, and subsequently TN^T^DNGR‐1, are not active against the latest emerged VOC (omicron and subsequent emerging linages) because of its V_HH_s binding limitations, TN^T^ scaffold provides efficient epitope accessibility for biparatopic V_HH_‐tandem assuring a broad and efficient neutralization of mutational variants, superior to other previously described trimeric or multimeric designs.^[^ ^31^, ^61^ ^]^

TN^T^DNGR‐1 shows specific binding to mouse BMDC cDC1s and pDCs, but not to the DNGR‐1‐negative cDC2 subset in vitro, mediating RBD delivery and internalization into cDC1 DNGR‐1‐positive cells. Remarkably, TN^T^DNGR‐1 contributes to the activation of adaptive immunity in vivo by the specific targeting of captured SARS‐CoV‐2 S protein towards cDC1s. In a prime‐boost immunization experiment in immunocompetent C57BL/6 mice, the coadministration of S protein and TN^T^DNGR‐1 resulted in a significant increase in the frequency and number of S‐specific effector CD8^+^ T cells and suggest a more Th1‐polarized antibody profile response, compared to the administration of protein S alone or coadministered with TN^T^. Furthermore, our results emphasize that the neutralizing capacity of an antibody is not sufficient for effective protection when applied therapeutically, with interactions with immune system receptors, such as FcγRs or DNGR‐1 on DCs or other antigen‐presenting cells, being essential for the development of effective immunity and disease control.^[^ ^51^, ^52^ ^]^ In this sense, even with exceptional neutralizing activity in vitro, the absence of effective interactions with immune receptors would explain why TN^T^ did not prevent viral spread in the lungs, elicit virus‐specific adaptative immune responses or prevent the death of mice. The protective effect against lethal SARS‐CoV‐2 challenge is rescued for TN^T^DNGR‐1 by targeting neutralized virions to cDC1, triggering their clearance and promoting adaptive immune responses, specifically CTL activity.

Accordingly, TN^T^DNGR‐1 also reduced virus load in the lungs, indicating that DNGR‐1‐targeting provides an effective control of the infection spread as conventional Fc‐containing nAbs.^[^ ^50^, ^51^, ^52^, ^62^ ^]^ TN^T^DNGR‐1 induced an early and efficient S‐specific IgM and S‐, RBD‐, and N‐specific IgG antibody response, while TN^T^ treatment did not increase endogenous antibody induction over non‐treated animal levels. The superior serum neutralizing activity at day 5 p.c. for the sotrovimab group, relates with its higher serum concentration compared to TN^T^DNGR‐1 and TN^T^ (8.26 µg mL^−1^, 12.72 and 0.32 ng mL^−1^, respectively), because of the longer half‐life of IgG,^[^ ^44^ ^]^ and does not indicate a higher level of induction of endogenous nAbs. By day 20 p.c. the induction of endogenous nAbs shows a potent effect for both TN^T^DNGR‐1 and sotrovimab treatment groups.

Finally, TN^T^DNGR‐1 and sotrovimab induced a significant increase in S protein‐specific CD4^+^ and CD8^+^ T cells in the lungs at day 20 p.c. But, while both treatments elicited similar levels of S‐specific CD4^+^ T cells, TN^T^DNGR‐1‐treatment specifically triggered a higher magnitude of S‐specific CD8^+^ T cells, characterized by secretion of IFNγ, TNFα, and IL‐2 or CD107a expression. The TN^T^DNGR‐1 protective effect, via cDC1s targeting through DNGR‐1 receptor, appears superior to that observed with a conventional neutralizing IgG, previously described to be mediated by CCR2^+^ monocytes, as well as cytotoxic CD8^+^ T cells infiltrating the lung.^[^ ^51^ ^]^ Taken together, in the context of a respiratory viral infection, TN^T^DNGR‐1 elicits efficient humoral responses and suggest an improved effect on local expansion of virus‐specific CD8^+^ T cells due to its alternative mechanism of action. In summary, we have generated a bispecific trimeric antibody that, in addition to its potent and broad SARS‐CoV‐2 neutralization activity, delivers virus antigens or whole virions to cDC1s in an Fc‐independent manner, priming systemic virus‐specific humoral and local CD4^+^ and CD8^+^ T cell responses in vivo. The therapeutical administration of TN^T^DNGR‐1 antibody in SARS‐CoV‐2‐infected K18‐hACE2 mice provided full protection against SARS‐CoV‐2 morbidity and mortality, causing reduced viral load in the lungs and increased S‐specific humoral and CD4^+^ and CD8^+^ T cell immune responses. Therefore, the strategy provides a bridge between passive immunotherapy with nAbs and a DC vaccination‐like action for the induction of enhanced adaptive immunity without the involvement of FcγRs. Our results indicate that TN^T^DNGR‐1is a promising candidate for the development of improved viral treatments, and readily adaptable to other SARS‐CoV‐2 VOCs or other viruses, given the modular nature of the molecule.

## 4 Conclusions

In summary, our study addresses a novel strategy based on a broadly neutralizing bispecific anti‐SARS‐CoV2 antibody that targets virions to type 1 conventional DCs (cDC1s) for promoting in vivo T cell cross‐priming. The strategy was shown to be therapeutically effective in protecting K18‐hACE2 mice from a lethal viral challenge while enhancing protein S‐specific CD8^+^ T cell responses.

## 5 Experimental Section

### Cell Lines

HEK‐293 (# ACC‐305, DSMZ) and 293T (# CRL‐3216, ATCC) cells were obtained from the ATCC and cultured in Dulbecco's Modified Eagle Medium (DMEM) (Lonza) supplemented with 2 mmol L^−1^ l‐glutamine (Gibco), 10% v/v heat‐inactivated fetal bovine serum (FBS) (Merck Life Science), and antibiotics (100 units mL^−1^ penicillin, 100 mg mL^−1^ streptomycin; Life Technologies) referred as to DMEM complete medium (DCM) As previously described, the parental B3Z cell line^[^ ^63^ ^]^ and its derived mouse cell line expressing CLEC9A/DNGR‐1 (B3Z^DNGR‐1^) were cultured in Roswell Park Memorial Institute (RPMI) medium (Gibco) supplemented with 2 mmol L^−1^ l‐glutamine, 10% v/v heat‐inactivated FBS, antibiotics (100 units mL^−1^ penicillin, 100 mg mL^−1^ streptomycin), herein referred as RCM, and 1 mmol L^−1^ sodium pyruvate (Gibco), 0.1 mmol L^−1^ MEM nonessential amino acids (NEAA) (Gibco), 55 pmol L^−1^ β‐mercaptoethanol (Gibco), and. Generation and characterization of the B3Z^DNGR‐1^ cell line was previously described.^[^ ^40^, ^64^ ^]^ BHK‐21/WI‐2 (# EH1011, Kerafast) cells, used for recombinant VSV generation, were grown in DCM. Vero E6 (# 60 476, BCRC) cells used in the S‐pseudotyped neutralization experiments were grown in DCM supplemented with 0.1 mmol L^−1^ NEAA and 12.5 units mL^−1^ Nystatin (penicillin‐streptomycin‐nystatin) (Biological Industries). Vero E6 cells expressing the transmembrane serine protease TMPRSS2 (VeroE6/TMPRSS2) used in the live SARS‐CoV‐2 neutralization experiments were maintained in DCM, supplemented with 10 mM HEPES (Gibco), 100 mg mL^−1^ geneticin (G 418 disulfate salt, Sigma‐Aldrich) and 0.1 mmol L^−1^ NEAA. Mouse BMDCs were obtained by using Flt3‐Ligand (Flt3‐L BMDCs) as previously described^[^ ^37^ ^]^ with slight modifications. Briefly, femur and tibia from C57BL/6J mice were collected, bone marrow was flushed, red blood cells were lysed and cells were cultured in RCM 1000 units mL^−1^ penicillin, 100 µg mL^−1^ streptomycin (both from Sigma‐Aldrich), 0.1 mmol L^−1^ NEAA, 1 mmol L^−1^ sodium pyruvate, 2 mmol L^−1^ l‐glutamine, 10 mmol L^−1^ HEPES (all from HyClone), and 50 µmol L^−1^ β‐mercaptoethanol (Merck), herein called R10, plus 200 ng mL^−1^ human Flt3‐L (Miltenyi). Media was refreshed on day 7 and cells were harvested on day 9. Mutu‐DC cells (kindly provided by Hans Acha‐Orbea, Laussane, Switzerland)^[^ ^65^ ^]^ were cultured in R10 and passaged when confluent using 5 mmol L^−1^ EDTA. All adherent cell lines were cultured at 37 °C in 5% v/v CO_2_ at 95% air in a humidified atmosphere. FreeStyle 293F and Expi293F (both from Gibco;) cells were respectively cultured in FreeStyle 293 and Expi293 Expression Medium at 37 °C in a humidified 8% CO_2_ incubator rotating at 95 rpm. All cell lines were used within three months of thawing and checked for Mycoplasma by PCR every month using the Mycoplasma Gel Detection Kit (Biotools B&M Labs).

### SARS‐CoV‐2 Viruses

Several SARS‐CoV‐2 viruses were used in the live SARS‐CoV‐2 neutralization assays. SARS‐CoV‐2 MAD6 isolate, a prototypic strain B.1 containing the D614G mutation in the S protein, is a virus collected from a nasopharyngeal swab from a 69‐year‐old male COVID‐19 patient from Hospital 12 de Octubre in Madrid, Spain.^[^ ^66^ ^]^ The VOCs B.1.351 (beta; hCoV‐19/France/PDL‐IPP01065/2021, clade 10H/501Y.V2) and B.1.617 (delta; SARS‐CoV‐2, Human, 2021, Germany ex India, 20A/452R) were supplied through the European Virus Archive Global (EVAg) platform, by the National Reference Centre for Respiratory Viruses hosted by Institut Pasteur (Paris, France) and the Robert Koch Institute (German Federal Institute for Infectious and Non‐Communicable Diseases, Berlin, Germany), respectively. All SARS‐CoV‐2 virus stocks were grown on Vero E6 cells and virus infectivity titers were determined by standard plaque or median tissue culture infectious dose (TCID_50_) assays as previously described.^[^ ^67^ ^]^

### Mouse Strains

C57BL/6 mice (Charles River Laboratories) were bred at CNIC under specific pathogen‐free conditions. Age‐matched female 6‐ to 8‐week‐old mice were used and randomized before treatment. Immunogenicity experiments in C57BL/6 mice were approved by the animal ethics committee at CNIC and by the Division of Animal Protection of the Comunidad de Madrid (PROEX 240/16). Transgenic K18‐hACE2 mice (The Jackson Laboratory), expressing hACE2 under control of the cytokeratin 18 promoter on the C57BL/6 background, were used in the protective efficacy studies. 9‐week‐old female K18‐hACE2 mice were used and randomized between treatment groups after SARS‐CoV‐2 challenge. Protective efficacy experiments with K18‐hACE2 mice were performed in the biosafety level 3 (BSL‐3) facilities at the Centro de Investigación en Sanidad Animal (CISA)‐Instituto Nacional de Investigaciones Agrarias (INIA)‐CSIC (Valdeolmos, Madrid, Spain). Efficacy experiments were approved by the animal ethics committee at INIA and by the Division of Animal Protection of the Comunidad de Madrid (PROEX 161.5/20). All animal procedures were conformed to Spanish law under the Royal Decree (RD 53/2013) and in accordance with EU Directive 2010/63EU and Recommendation 2007/526/EC.

### Production and Purification of the SARS‐CoV‐2 S and RBD Proteins

The SARS‐CoV‐2 HexaPro S protein cDNA,^[^ ^68^ ^]^ corresponding to Wuhan‐Hu‐1 strain, was codon‐optimized and synthesized (GenScript). The gene was cloned into mammalian expression vector pcDNA3.1(−) carrying a C‐terminal Twin‐Strep tag. Four days following the transfection in FreeStyle 293F cells with 293fectin transfection reagent (Life Technologies), the culture supernatants were collected and purified with Strep‐Tactin affinity chromatography resin (IBA LifeSciences) followed by SEC using a Superose 6 Increase 10/300 GL column (Cytiva). Concentrated purified fractions were analyzed by 0.1% w/v sodium dodecyl sulfate (SDS)−4–20% w/v polyacrylamide gel electrophoresis (PAGE). Proper protein folding and conformation was certified by antibody recognition in ELISA using anti‐RBD (# A2286, Biovision), anti‐N‐terminal domain (NTD) (# A2269, Biovision) and anti‐S2 (# 40590‐D001, SinoBiological) mAbs.

The SARS‐CoV‐2 Wuhan‐Hu‐1/B.1 strain S protein RBD, defined as amino acids R328‐L533, was expressed in pcDNA3.1(+) with an N‐terminal mu‐phosphatase signal peptide and a C‐terminal octa‐histidine tag (BEI Resources NR‐52422).^[^ ^14^ ^]^ The RBD was produced using Expi293F transfection, supernatant was harvested after 3 d, and protein was purified using a 10 mL bed volume of Talon Superflow Metal Affinity Cobalt Resin (Takara Bio). Purified protein was filtered and concentrated into buffer containing 20 × 10^−3^ m Tris, 300 × 10^−3^ m NaCl, and 200 × 10^−3^ m imidazole, pH 8.0. SDS‐PAGE was run to check purity and a modified competition ELISA as control for binding to hACE2 (Acro BioSystems).

### Generation of Antibody Expression Vectors

DNA fragments encoding for E, V, and TN (EV‐tandem) nanobodies were synthesized by GeneArt (Thermo Fisher Scientific) including them within the following vectors: pcDNA3.1(+)‐E V_HH_‐Myc‐His, pcDNA3.1(+)‐V V_HH_‐Flag‐His and pcDNA3.1(+)‐TN‐StrepTagII (Key Resources Table). All constructs included an in frame fused wild type N‐terminal interleukin‐2 (IL‐2) signal peptide for effective protein secretion to the medium. The expression vector for the TN^T^ trimerbody was generated by subcloning L23‐TIE cDNA fragment from vector pCR3.1 (+)‐MFE23scFv‐L23‐TIE‐Myc‐His (previously described^[^ ^43^ ^]^) into pCR3.1‐TN‐StrepTagII by mean of *Not*I/*BamH*I restriction enzymes. To generate the TN^T^DNGR‐1 construct, the cDNA for L15‐7H11scFv‐StrepTagII fragment (GeneArt) was subcloned using *Bgl*II/*Xba*I cloning sites into the TN^T^ trimerbody parental vector resulting in pcDNA3.1(+)‐TN‐L23‐TIE‐L15‐7H11‐StrepTagII. All the sequences were verified using primers FwCMV and RvBGH (Key Resources Table).

### Production and Purification of Recombinant Engineered Antibodies

The generation of HEK‐293 stable transfectants was done in DCM by selection with 500 mg mL^−1^ G418 (Life Technologies) after Lipofectamine 3000 (Invitrogen) transfection. Large‐scale protein production was done by collecting periodically the media of stable transfectants. TN, TN^T^, and TN^T^DNGR‐1 were purified from conditioned culture media by Strep‐Tactin affinity chromatography in an AKTA Prime Plus FPLC System (Amersham Biosciences). Finally, the elution fractions corresponding to purified antibodies were dialyzed against PBS. Protein concentration calculation was carried out by UV‐absorbance measurements on an Uvikon 930 spectrophotometer (Kontron Instruments). Purified antibodies were analyzed by 0.1% w/v SDS‐15% w/v PAGE in reducing conditions followed by Coomassie brilliant blue R‐250 (BioRad Laboratories) staining, for optimal band visualization. Gel image acquisition was performed with a ChemiDoc MP Imaging System (BioRad Laboratories).

### ELISA Assays

The ability of TN based constructs to bind purified SARS‐CoV‐2 RBD was analyzed by ELISA. Briefly, Nunc MaxiSorp flat‐bottom 96‐well plates (Thermo Fischer Scientific) were coated (0.2 µg/well) with recombinant B.1 RBD. After washing and blocking with 300 µL PBS‐BSA, 100 µL of conditioned media from transfected HEK‐293 cells were added to the wells and incubated for 1 h at room temperature. After three washes, 100 µL of the corresponding anti‐Strep mAb (# 2‐1507‐001, IBA Lifesciences) were added for 1 h at room temperature. Finally, HRP‐conjugated GAM IgG (# 115‐035‐003, Jackson ImmunoResearch Labs) was added, after which the plate was washed again and developed. Purified TN and TN^T^ (50, 10, and 1 × 10^−9^ m) ability to bind different strain RBDs was performed in the same way. RBD interaction with biotinylated hACE2 (# Q9BYF1‐1, AcroBiosystems) was used as control by adding 100 µL at 0.1 µg mL^−1^ for 1 h, followed by HRP‐conjugated streptavidin (# 554 066, BD Biosciences) in the same conditions. All optical density measurements were done using a Multiskan FC apparatus (Thermo Scientific Scientific).

### Biolayer Interferometry

The binding of TN and TN^T^ to immobilized B.1 or B.1.351 (beta) RBDs was measured using biolayer interferometry (BLI) on an Octet RED96 system (Fortebio). The RBD was immobilized onto AR2G biosensors (Fortebio) at pH 5.0 using a standard amine reactive coupling protocol (activation with EDC/s‐NHS and quenching with ethanolamine). The antibodies in HEPES‐buffered saline (HBS; 20 × 10^−3^ m HEPES, 150 × 10^−3^ m NaCl, pH 7.4) at 50 and 10 × 10^−9^ m were associated with the RBD for 30 min, after which the dissociation of antibody from the biosensor was measured for 1 h, to determine their binding kinetics. The experimental data was then fitted to a 1:1 binding model using the Octet Data Analysis software (Fortebio). To determine the blocking of ACE2's RBD binding, 150 × 10^−9^ m of TN monomer or 50 × 10^−9^ m of TN^T^ were associated with immobilized B.1 RBD for 10 min, after which 100 × 10^−9^ m of soluble hACE2 (Acro Biosystems) was added and associated with RBD for 30 min. To investigate bispecific interactions between the TN^T^DNGR‐1, immobilized RBD and DNGR‐1 in solution, RBD‐coated biosensors were first prepared as described above. Then, 50 × 10^−9^ m of the TN^T^DNGR‐1 in HBS was then incubated with the biosensors for 30 min, followed by 5 min of dissociation in HBS. The biosensors were then moved into 50 × 10^−9^ m of mouse DNGR‐1 Fc chimera in HBS and incubated for 30 min, followed by 5 min of dissociation.

### SEC‐MALS Experiments

Static light scattering experiments were performed on a Superdex 200 Increase 10/300 GL column (Cytiva) attached in‐line to a DAWN EOS light scattering photometer and an Optilab rEX differential refractive index detector (both from Wyatt Technology). The chromatography was run at room temperature and the scattering detector was thermostated at 23 °C. The column was equilibrated with running buffer (PBS pH 7.4 plus 150 × 10^−3^ m NaCl, 0.1 µm filtered) and the SEC‐MALS system was calibrated with a sample of BSA at 2 mg mL^−1^ in the same buffer. ≈150 µL of the solutions at 0.5 mg mL^−1^ were injected into the column at a flow rate of 0.5 mL min^−1^. Data acquisition and analysis were performed using ASTRA software (Wyatt Technology). The reported molar masses correspond to the center of the chromatography peaks. Based on numerous measurements on BSA samples under similar conditions it was estimated that the experimental error in the molar mass is around 5%.

### Circular Dichroism

Circular dichroism (CD) measurements were performed with a Jasco J‐715 spectropolarimeter (JASCO). The spectra were recorded on protein samples at 0.02 mg mL^−1^ in PBS pH 7.4 plus 150 × 10^−3^ m NaCl using a 0.2 cm path length quartz cuvette at 25 °C. Thermal denaturation curves from 10 to 95 °C were recorded on the same protein samples and cuvette by increasing temperature at a rate of 1 °C min^−1^ and measuring the change in ellipticity at 218 nm.

### Microfluidic Diffusional Sizing

The change in hydrodynamic radius (*R*_h_) of TN^T^ trimerbody and its subsequent complex with SARS‐CoV‐2 S protein was measured by microfluidic diffusional sizing (MDS).^[^ ^69^ ^]^ Then, *R*_h_ values of fluorescently labeled species in their native state in solution are included into a plot of the hydrodynamic radii of a range of native and denatured standard proteins obtained using dynamic light scattering and pulsed field gradient nuclear magnetic resonance.^[^ ^70^ ^]^ TNT (1 × 10^−6^ m) was labelled with Fluidiphore rapid amine 503 through amine coupling. The sample was incubated at 4 °C overnight and the size of the conjugated protein was determined through measuring the hydrodynamic radius, *R*_h_, using the Fluidity One‐W platform. To determine the *R*_h_ upon complex formation, the unlabeled HexaPro S protein (1 × 10^−6^ m) was incubated 1:1 with labeled TN^T^ for 5 min. The change in *R*_h_ was monitored by Fluidity One‐W instrument.

### Cryo‐EM

A sample of 0.5 mg mL^−1^ HexaPro S protein was prepared in the protein production core (C1) of the Center for Biomolecular Therapeutics (Rockville, Maryland, USA), as previously described.^[^ ^71^ ^]^ It was mixed in 1:1 molar ratio with TN^T^ trimerbody and vitrified on cryo‐EM grids using standard protocols with an FEI Vitrobot Mark IV.  Images were collected on an FEI 200 kV Glacios electron microscope equipped with a Gatan K3 direct electron detector and processed in cryoSPARC (data collection statistics are shown in Figure S5F in the Supporting Information). Particles were selected using blob picking and these were used to produce an ab initio model that was quickly refined and used to generate particle templates for repicking and 2D classification/ filtering. Multiple cycles of 3D refinement in cryoSPARC^[^ ^72^ ^]^ resulted in electron density maps at 3.80 Å resolution as determined from gold‐standard Fourier shell correlation (GSFSC) curves. This model was in excellent agreement with overall structure of the S protein; however, density regions corresponding to bound TN^T^ elements were difficult to resolve likely due to heterogeneity and dynamic nature of the protein complex. To improve the quality and interpretability of the electron density, particle stacks were exported into Relion^[^ ^73^ ^]^ and subjected to 3D refinement with imposed C3 symmetry followed by 3D classification without any symmetry to allow determination of subconformations not compliant with C3 symmetry. This produced 3 distinct subconformations that were subjected to a single final round of non‐uniform refinement in cryoSPARC and local map sharpening in PHENIX.^[^ ^73^ ^]^

### Cryo‐EM Model Building and Refinement

Model building on the map resulting after the incubation of TN^T^ with the HexaPro S protein was done in Coot 0.9.6.^[^ ^74^ ^]^ The coordinates corresponding to PDB 7B18^[^ ^23^ ^]^ were used as a starting model, placed into it after performing a molecular replacement step and posterior refinement in PHENIX,^[^ ^75^ ^]^ that included rigid body, simulated annealing and morphing. The model was manually fitted into the density sharpened map and several runs of refinement done in PHENIX until a final reliable model were obtained. Figures were generated using ChimeraX.^[^ ^76^ ^]^

### Flow Cytometry Studies

The ability of both TN‐based trimerbodies to bind SARS‐CoV‐2 B.1 S protein on cell surface was studied by flow cytometry. HEK‐293 or HEK‐293^S^ cells were incubated with supernatants or purified trimerbodies (1 µg mL^−1^) followed by anti‐Strep mAb and PE‐GAM IgG antibody (# 115‐116‐071, Jackson ImmunoResearch Labs) for 30 min at 4 °C, each of them. Between incubations and after the final one, the cells were sedimented by centrifugation (1200 g, 4 °C, 5 min) and washed with PBS supplemented with 0.05% v/v FBS (PBS‐FBS) for three times. As a control, both cell types were incubated with rabbit anti‐SARS‐CoV‐2 IgG SB‐40592 (# 40592‐R001, Sino Biological) followed by AF488‐conjugated donkey anti‐rabbit IgG (# 711‐545‐152, Jackson ImmunoResearch Labs). The binding of TN^T^DNGR‐1 to cell surface mouse CLEC9A/DNGR‐1 was assayed using parental B3Z and transfected B3Z^DNGR‐1^ cell lines following the same protocol and staining as explained above for S protein labelling. The PE‐conjugated rat anti‐human Clec9A (CD370) antibody (clone 3A4/Clec9A) (# 563 488, BD Biosciences) was used as control. Furthermore, the capacity of TN^T^DNGR‐1 for targeting soluble RBD specifically to B3Z^DNGR‐1^ cells was analyzed by preincubating AF647‐conjugated RBD (1 µg mL^−1^) with TN^T^DNGR‐1 or TN^T^ (1 µg mL^−1^) for 30 min at room temperature. Then, the mixtures were added to the cells and followed by incubation of 30 min at 4 °C. All the samples were processed on a FACScan apparatus (BD Biosciences) while data analysis was done using the FlowJo Software (BD Biosciences). For labeling and endocytosis studies in BMDCs, cells were incubated with culture media containing TN^T^ or TN^T^DNGR‐1 (1× supernatant and 10×, respectively) for 30 min at 4 °C. Cells were then washed and kept for different times at 37 °C or 4 °C before being incubated with AF647‐conjugated RBD (1 µg mL^−1^) for 1 h at 4 °C and further stained with antibodies to distinguish the main DC subpopulations: type 1 DCs (cDC1s, CD11c^+^B220^−^XCR1^+^), type 2 DCs (cDC2s, CD11c^+^B220^−^SIRPa^+^), and pDCs (CD11c^+^B220^+^). Stained cells were analyzed on BD FACSCanto flow cytometer (BD Biosciences).

### Confocal Microscopy

MuTuDC cells were incubated with culture media containing TN^T^ or TN^T^DNGR‐1 (1× supernatant and 10×, respectively) for 30 min at 4 °C, washed and stained with AF647‐conjugated RBD (1 µg mL^−1^) for 1 h at 4 °C. Cells were then thoroughly washed and incubated in complete media for different times at 4 or 37 °C. Then, stained cells were resuspended in PBS, plated in Cell‐Tak (Corning) coated coverslips, fixed using 4% paraformaldehyde (PFA) and mounted for visualization in a Zeiss LSM 700 confocal microscope.

### Production of SARS‐CoV‐2 S‐Pseudotyped VSV Particles

Vesicular stomatitis virus‐G (VSV‐G) pseudotyped rVSV‐luc recombinant viruses were produced according to previously published protocol by cotransfection of BHK‐21 cells (BHK‐21/WI‐2, Kerafast) with expression vector coding for VSV‐G and rVSVΔG‐luciferase vector (G*ΔG‐luciferase; Kerafast) that contains firefly luciferase instead of the VSV‐G open reading frame.^[^ ^77^ ^]^ Briefly, BHK‐21 cells were transfected using Lipofectamine 3000 protocol to express the SARS‐CoV‐2 S protein and after 24 hours, the transfected cells were inoculated with a replication‐deficient VSV‐G‐rVSV‐luc pseudotype (multiplicity of infection (MOI) of 3). Following 1 h incubation at 37 °C, the inoculum was removed, cells were washed extensively with PBS and fresh DCM was added. SARS‐CoV‐2 S‐pseudotyped rVSV‐luc particles were harvested 20 h postinoculation, clarified from cellular debris by centrifugation and stored at −80 °C. Infectious titers were estimated as TCID_50_ mL^−1^ by limiting dilution of the SARS‐CoV‐2 S rVSV‐luc‐containing supernatants on Vero E6 cells. Luciferase activity was determined by Steady‐Glo Luciferase Assay System in a GloMax Navigator Microplate Luminometer (both from Promega). The SARS‐CoV‐2‐pseudotyped rVSV‐luc variants used were SARS‐CoV‐2 S protein B.1, alpha, beta, gamma, delta, and kappa. The SARS‐CoV‐2 S protein mutant D614G (B.1) was generated by site‐directed mutagenesis using as an input DNA the expression vector encoding SARS‐CoV‐2 S 614D by Q5 Site Directed Mutagenesis Kit (New England Biolabs) following the manufacturer's instructions. SARS‐CoV‐2 alpha (B.1.1.7; GISAID: EPI_ISL_608 430), beta (B.1.351; GISAID: EPI_ISL_712 096), gamma (P.1; GISAID: EPI_ISL_833 140), delta (B.1.617.2; GISAID: EPI_ISL_1 970 335), and kappa (B.1.617.1; GISAID: EPI_ISL_1 970 331) were optimized, synthesized and cloned into pcDNA3.1 (GeneArt).

### SARS‐CoV‐2 S Protein‐Pseudotyped VSV Neutralization Assay

TN and TN^T^ neutralization activity were examined in triplicates at concentrations in the range 0.64 × 10^−12^ m – 10 × 10^−9^ m using a SARS‐CoV‐2‐pseudotyped rVSV‐luc system. As controls, the SB‐40592 anti‐RBD neutralizing mAb and sotrovimab biosimilar (anti‐S glycoprotein mAb) were used in the range 0.88 × 10^−12^ m – 13.86 × 10^−9^ m. For neutralization experiments, viruses‐containing transfection supernatants were normalized for infectivity to a MOI of 0.5‐1 and incubated with the antibodies at 37 °C for 1 hour in 96‐well plates. After the incubation time, 2 × 10^4^ Vero E6 cells were seeded onto the virus‐antibody mixture and incubated at 37 °C for 24 h. Cells were then lysed and assayed for luciferase expression. Half maximal effective concentration (EC_50_) and 95% confidence intervals (95% CI) were calculated using a nonlinear regression model fit with settings for log agonist versus normalized response curve using GraphPad Prism v8 Software.

### Live SARS‐CoV‐2 Neutralization Assay

The neutralization activity of TN‐based trimerbodies was also measured by a microneutralization test assay by using live SARS‐CoV‐2 virus, as previously described.^[^ ^67^ ^]^ Serially five‐fold diluted antibodies (in the range 0.3 × 10^−12^ m – 5 × 10^−9^ m) in DMEM‐2% v/v FBS medium were incubated at a 1:1 ratio with 200 TCID_50_ of live SARS‐CoV‐2 B.1 (MAD6 isolate, containing the D614G mutation), B.1.351 (beta) and B.1.617.2 (delta) VOCs in U‐bottom 96‐well tissue culture plates for 1 h at 37 °C. Then, mixtures of antibodies and live SARS‐CoV‐2 virus were added in triplicates to Vero E6/TMPRSS2 cell monolayers seeded in 96‐well plates at 2 × 10^4^ cells/well, and plates were incubated at 37 °C, in a 5% CO_2_ incubator for 3 d. Then, cells were fixed with 10% formaldehyde for 1 h and stained with crystal violet. When plates were dried, crystal violet was diluted in H_2_O‐10% w/v SDS and optical density was measured in a luminometer at 570 nm. Fifty percent neutralization titer (NT_50_) and 95% CI were calculated using a nonlinear regression model fit with settings for log agonist versus normalized response curve using GraphPad Prism v8 Software.

### Immunization of Immunocompetent C57BL/6 Mice

Trimerbodies in combination with SARS‐CoV‐2 S protein super stable trimer (Acro Biosystems) were injected subcutaneously (s.c.) into both hind paws of C57BL/6 mice. The immunization followed a prime‐boost scheme 7 days apart. Mice (*n* = 5/group, but PBS *n* = 3/group) were primed either with (1) PBS as control; (2) 2 µg of S protein alone; (3) 3 µg of TN^T^ + 2 µg of S protein; or (4) 3 µg of TN^T^DNGR‐1 + 2 µg of S protein. For the boost, mice were injected s.c. respectively with (1) PBS as control; (2) 1 µg of S protein alone; (3) 1.5 µg of TN^T^ + 1 µg of S protein; or (4) 1.5 µg of TN^T^DNGR‐1 + 1 µg of S protein. These amounts were divided equally between the two hind paws. All conditions except PBS included 5 µg CpG ODN 1826 and 5 µg Poly (I:C) LMW (both from InvivoGen) per mouse as adjuvants. The injection volume was 25 µL per paw. Mice were euthanized on day 14 (7 d post‐boost) and spleens and popliteal lymph nodes (pLNs) were harvested to assess cellular responses by flow cytometry analysis, and serum samples were collected to evaluate humoral responses.

### Cellular Response Analysis of Immunized C57BL/6 Mice

Spleens and pLNs were smashed through a 70 µm filter in R10 medium. In spleen suspensions, erythrocytes were lysed using 1 mL of red blood cell lysing buffer Hybri‐Max (Sigma‐Aldrich). Cells were stained with fluorochrome labelled antibodies directed against B220‐FITC (# MABF608, Invitrogen), CD11c‐BV421 (# 566 877, BD Biosciences), CD11c‐APC/Fire (# 117 351, BioLegend), CD19‐BV421(# 302 233, BioLegend), CD8‐APC/Fire (# 100 765, BioLegend), CD4‐APC (# 553 051, BD Biosciences), CD44‐FITC (#11‐0441‐82, Invitrogen), Sirpa‐PE/Cy7 (# 25‐1721‐82, Invitrogen), and XCR1‐PE (# 148 203, Biolegend), CD16/CD32 Fc Shield (# 70‐0161, Tonbo Biosciences) was used to reduce nonspecific binding. All antibodies were used at a 1:200 dilution. Tetramer specific for SARS‐CoV‐2 S protein, H‐2Kb (^539^VNFNFNGL^546^), was provided by the NIH Tetramer Facility at Emory University (1:100 dilution). Samples were stained in cold PBS supplemented with 2.5% v/v FBS and 2 mM EDTA. Tetramer staining was performed at room temperature for 15 min, followed by sequential surface staining with the appropriate antibody cocktail at 4 °C for 15 min. Dead cells were excluded using DAPI. Data was acquired on a LSRFortessa Cell Analyzer (BD Biosciences), using FACSDiva software, and analyzed using FlowJo v10.8 Software (BD Biosciences).

### Humoral Response Analysis of Immunized C57BL/6 Mice

Serum samples were prepared by incubating blood samples in collection tubes without anticoagulant for 15 min at room temperature to allow clotting to occur. Then samples were centrifuged at 15,000 rpm for 15 min, and serum was moved to a fresh collection tube. S‐ and RBD‐specific immunoglobulin (Ig) levels were determined by ELISA. Briefly, SARS‐CoV‐2 S protein or RBD were plated in Nunc MaxiSorp flat‐bottom 96‐well plates (Thermo Fischer Scientific) in carbonate buffer or PBS, respectively, at 1 µg mL^−1^ overnight at 4 °C. Then, the plates were washed three times with PBS and blocked with BSA‐PBS at least 1 h at 37 °C. After washing again, serum was plated by duplicate at 1/50, 1/250, 1/1250, and 1/6250 serial dilutions in PBS containing 1% w/v BSA to visualize dose‐response signals. Samples were incubated 2 h at room temperature and washed three times with PBS‐0.05% v/v Tween‐20. Plates containing serum samples were incubated with biotinylated anti‐mouse IgM, total IgG, IgG1, and IgG2c (all from BD) at 2 µg mL^−1^ for 1 h at room temperature followed by three washes with PBS‐0.05% (v/v) Tween‐20 and incubation with HRP‐conjugated streptavidin at 1 µg mL^−1^ for 30 min at room temperature. All plates were then washed again and developed in the presence of 3,3′,5,5′‐Tetramethylbenzidine (TMB) substrate (Sigma‐Aldrich) and stopped by 1 m H_2_SO_4_ solution. Absorbance was read at 450 nm subtracting background at 620 nm.

### Protective Therapeutic Efficacy Study in Transgenic K18‐hACE2 Mice

Female K18‐hACE2 mice (9‐weeks‐old) were used to evaluate the capacity of TN^T^ and TN^T^DNGR‐1 antibodies to protect against SARS‐CoV‐2 infection in a therapeutic administration schedule. After a lethal dose challenge (1 × 10^5^ PFUs/mouse) with SARS‐CoV‐2 (MAD6 strain) by intranasal route in 50 µL of PBS, mice were divided randomly in four groups (*n* = 8/group) and 17 h later were inoculated i.p. with 100 µg of TN^T^DNGR‐1, 65 µg of TN^T^ (equimolar to TN^T^DNGR‐1), 100 µg of sotrovimab or PBS (as a control of infection) in a total volume of 200 µL. In addition, noninfected and nontreated mice (*n* = 8) were used as a control group of healthy animals.

Mice were monitored for body weight change and mortality for 20 d p.c. Animals with more than 25% of body weight loss were euthanized. At days 5 and 20 p.c., four mice per group were euthanized, and lungs and serum samples were collected. Right lung lobes were divided longitudinally in two, with one part placed in RNALater stabilization reagent (Sigma‐Aldrich) and stored at −80 °C for RNA extraction, and the other part stored also at −80 °C for the analysis of infective virus yields. At day 20 p.c. left lung was freshly processed for cellular immune response analysis. At both end point times blood was collected by submandibular bleeding, maintained at 37 °C for 1 h, kept at 4 °C overnight, and centrifuged at 3600 rpm for 20 min at 4 °C to obtain the serum samples, which was then inactivated at 56 °C for 30 min and kept at −20 °C until use.

### Analysis of SARS‐CoV‐2 RNA by RT‐qPCR

Lungs from K18‐hACE2 mice were harvested at day 5 p.c. and stored in RNALater (Sigma‐Aldrich) at −80 °C until homogenized with a gentleMACS dissociator (Miltenyi Biotec) in 2 mL of RLT buffer (Qiagen) plus β‐mercaptoethanol (Sigma‐Aldrich) and aliquoted. Then, 600 µL of homogenized lung tissue was used to isolate total RNA using the RNeasy minikit (Qiagen), according to the manufacturer's specifications. First strand cDNA synthesis and subsequent real‐time PCR were performed in one step using NZYSpeedy One‐step RT‐qPCR Master Mix (NZYTech), according to the manufacturer's specifications using ROX as reference dye. SARS‐CoV‐2 viral RNA content was determined using previously validated set of primers and probes specific for the SARS‐CoV‐2 subgenomic RNA for the protein E and the genomic virus RNA dependent RNA polymerase (RdRp) gene.^[^ ^78^ ^]^ Data were acquired with a 7500 real‐time PCR system (Applied Biosystems) and analyzed with 7500 software v2.0.6. Relative RNA arbitrary units (A.U.) were quantified relative to a negative group (noninfected mice) and were performed using the 2^−ΔΔCt^ method. All samples were tested in duplicate.

### Analysis of SARS‐CoV‐2 Virus Yields by Plaque Assay

Lungs from K18‐hACE2 mice were harvested at day 5 p.c., weighted, and stored directly at −80 °C until homogenized with a gentleMACS dissociator (Miltenyi Biotec) in 2 mL of PBS and aliquoted. Then, undiluted and serial tenfold dilutions of homogenized lung tissue were added in triplicate to Vero E6/TMPRSS2 cell monolayers seeded in 12‐well plates at 5 × 10^5^ cells/well. After 1 h of adsorption the inoculums were removed and plates were incubated at 37 °C, 5% v/v CO_2_ in 2:1 DMEM 2× containing 4% FBS and Avicel RC‐591 (DuPont Nutrition Biosciences ApS). After 4 d, cells were fixed for 1 h with 10% formaldehyde (Sigma‐Aldrich), then the supernatant was removed, and plaques were visualized by adding 0.5% w/v crystal violet solution (Sigma‐Aldrich). SARS‐CoV‐2 titers were determined in PFUs per gram of lung tissue.

### Humoral Response Analysis in the Protective Therapeutic Efficacy Experiment in Transgenic K18‐hACE2 Mice

S‐specific IgM measurements in individual sera samples from SARS‐CoV‐2 infected and treated K18‐hACE2 mice were done by ELISA as described above in prime‐boost immunization assays. S‐, RBD‐, and N‐specific IgG antibody levels were determined by ELISA, as previously described.^[^ ^67^, ^78^, ^79^ ^]^ Briefly, 96‐well Nunc MaxiSorp plates were coated with 50 µL of purified recombinant SARS‐CoV‐2 S or RBD proteins (2 µg mL^−1^) in PBS overnight at 4 °C. The SARS‐CoV‐2 S and RBD proteins used to coat the plates derived from the Wuhan strain (GenBank accession number MN908947.3) and were previously described.^[^ ^67^, ^78^, ^79^ ^]^ Plates were washed with PBS‐0.05% v/v Tween‐20 and blocked with 5% milk in PBS for 2 h at room temperature. Individual serum samples were diluted in duplicate in PBS‐0.05% Tween‐1% milk, added to plates, and incubated for 1.5 h at room temperature. Plates were then washed, and secondary HRP‐conjugated GAM IgG mAb (Southern Biotech) diluted 1:1000 in PBS‐0.05% Tween‐1% milk was added and incubated for 1 hour at room temperature. Plates were washed, the TMB substrate (Sigma‐Aldrich) was added, and the reaction was stopped by adding 1 m H_2_SO_4_. Absorbance was read at 450 nm. Antigen‐specific IgG and IgM serum levels were evaluated by absorbance summation method, which sums the observed absorbance values from all dilutions employed to obtain one data point for each sample to be used for comparison.^[^ ^80^ ^]^

### Determination of Administrated Antibody Concentration in Serum

The concentration of TN^T^, TN^T^DNGR‐1 or sotrovimab in serum samples at day 5 and 20 p.c. was determined by ELISA using 1:5 serial dilutions of the corresponding purified antibodies for standard. Briefly, Nunc MaxiSorp flat‐bottom 96‐well plates (Thermo Fischer Scientific) were coated with recombinant B.1 RBD (0.2 µg/well). After washing and blocking with 300 µL PBS‐BSA, 50 µL of individual sera, 1/20 and 1/100 times diluted, were incubated for 1 h at room temperature. For TN^T^ and TN^T^DNGR‐1 or sotrovimab, after three washes, 100 µL of the corresponding HRP‐conjugated rabbit anti‐camelid V_HH_ cocktail (# A02016, Genescript) or HRP‐conjugated goat anti‐human IgG mAb (# A0170, Sigma‐Aldrich) were added, respectively, and incubated 1 h at room temperature. Finally, the plate was washed again and developed. All samples were analyzed by duplicate per each dilution factor.

### Cellular Response Analysis in the Protective Therapeutic Efficacy Experiment in Transgenic K18‐hACE2 Mice

Lung cellular suspensions were obtained from the left lung of K18‐hACE2 mice harvested at day 20 p.c., homogenized with a gentleMACS dissociator (Miltenyi Biotec) in R10 medium and then erythrocytes were lysed by using ACK lysis buffer (Gibco). S‐specific H‐2Kb tetramer staining was performed, acquired and analyzed as previously described for the C57BL/6 prime‐boost immunization assay. The magnitude, breadth and polyfunctionality of SARS‐CoV‐2 S‐specific CD4^+^ and CD8^+^ T cells expressing CD107a, and/or secreting IFNγ, and/or TNFα, and/or IL‐2 were analyzed by ICS as previously described^[^ ^78^, ^79^ ^]^ in lung cell samples stimulated with a SARS‐CoV‐2 S peptide pool (1 µg mL^−1^) (JPT Peptide Technologies), spanning the S1 and S2 regions of the S protein from the Wuhan strain, and containing 158 (S1) and 157 peptides (S2) as consecutive 15‐mers overlapping by 11 amino acids. After left lung processing, 4 × 10^6^ fresh isolated cells were seeded on M96 plates and stimulated overnight in complete RPMI 1640 medium supplemented with 10% FBS containing 1 µL mL^−1^ Golgiplug (BD Biosciences) to inhibit cytokine secretion, 1× monensin (eBioscience, Thermo Fisher Scientific), and anti‐CD107a–FITC (BD Biosciences). Cells were then washed, stained for surface markers, fixed, permeabilized (Cytofix/Cytoperm kit; BD Biosciences), and stained intracellularly with the appropriate antibodies. Dead cells were excluded using the violet LIVE/DEAD stain kit (Invitrogen). The fluorochrome‐conjugated antibodies used for functional analyses were CD3‐PE‐CF594, CD4‐APC‐Cy7, CD8‐V500, IFN‐γ–PE‐Cy7, TNF‐α–PE, and IL‐2–APC. All antibodies were from BD Biosciences. Cells were acquired with a Gallios flow cytometer (Beckman Coulter), and data analyzed with the FlowJo software version 10.4.2 (Tree Star), as previously described.^[^ ^78^, ^79^ ^]^ Gating strategy and example is shown in Figure S7C,D in the Supporting Information.

### Equipment and Settings

Gels and Western blot membrane images from Figures S2, S3, and S7 (Supporting Information) were acquired and analyzed using the ChemiDoc MP Imaging System and Image Lab analysis software (both from BioRad Laboratories). If brightness or contrast processing of gel and blot images has been performed, it was applied to the entire image, including controls. No high‐contrast gels or blots have been displayed. When necessary, cropped gels and juxtaposing images were displayed to improve the clarity and conciseness of the presentation, being shown in the figure. All optical density measurements were done using a Multiskan FC apparatus (Thermo Fischer Scientific).

### Statistical Analysis

Statistical analysis was performed using GraphPad Prism Software version 8.0. In general, the in vitro experiments were done in triplicates and values are presented as mean ± standard error of the mean (SEM) from one of at least two independent experiments. In the immunogenicity experiments in C57BL/6 mice, significant differences (*P* value) were discriminated by applying one‐way ANOVA followed by Tukey's multiple comparison test. The multiparametric read out analysis of protective efficacy experiment in K18‐hACE2 mice, in general was done by applying one‐way ANOVA followed by Tukey's multiple comparison test for significant differences discrimination.

## Conflict of Interest

L.A.‐V. and L.S. are co‐founders of Leadartis S.L., M.C., and R.N. are current employees of Leadartis S.L.

## Author Contributions

R.L.‐G., P.P., I.H.‐M., I.A.‐B., G.A., D.A., S.F., J.L., N.L., S.L.H., A.S.‐T., L.R.‐P., C.A., R.N., M.C., E.P., R.G.‐R., E.G.‐R. designed and performed the experiments and analyzed the data. R.L.‐G., P.P., I.H.‐M. and prepared the figures. X.S., Y.L., K.A.A., D.A., W.Y., A.D.M. helped with the antibody complex visualization and modeling. R.L.‐G., P.P., I.H.‐M., wrote the original draft. R.L.‐G. and L.A.‐V. prepared the manuscript. R.L.‐G., P.P., I.H.‐M., R.G.‐R., D.S., J.G.‐A., L.A.‐V. contributed to the experimental and conceptual design and edited the manuscript. L.A.‐V., J.G.‐A., D.S., M.E., R.D., I.G.M., L.S., D.J.W., F.J.B., E.P. supervised the project.

## Supporting information

Supporting Information

## Data Availability

The data that support the findings of this study are available on request from the corresponding author. The data are not publicly available due to privacy or ethical restrictions.

## References

[1] L. R. Baden , H. M. El Sahly , B. Essink , K. Kotloff , S. Frey , R. Novak , D. Diemert , S. A. Spector , N. Rouphael , C. B Creech , J. Mcgettigan , S. Khetan , N. Segall , J. Solis , A. Brosz , C. Fierro , H. Schwartz , K. Neuzil , L. Corey , P. Gilbert , H. Janes , D. Follmann , M. Marovich , J. Mascola , L. Polakowski , J. Ledgerwood , B. S. Graham , H. Bennett , R. Pajon , C. Knightly , et al., N. Engl. J. Med. 2021, 384, 403.33378609

[2] F. P. Polack , S. J. Thomas , N. Kitchin , J. Absalon , A. Gurtman , S. Lockhart , J. L. Perez , G. Pérez Marc , E. D. Moreira , C. Zerbini , R. Bailey , K. A. Swanson , S. Roychoudhury , K. Koury , P. Li , W. V. Kalina , D. Cooper , R. W. Frenck , L. L. Hammitt , Ö. Türeci , H. Nell , A. Schaefer , S. Ünal , D. B. Tresnan , S. Mather , P. R. Dormitzer , U. Sahin , K. U. Jansen , W. C. Gruber , N. Engl. J. Med. 2020, 383, 2603. 33301246 10.1056/NEJMoa2034577 PMC7745181

[3] M. Voysey , S. A. C. Clemens , S. A. Madhi , L. Y. Weckx , P. M. Folegatti , P. K. Aley , B. Angus , V. L. Baillie , S. L. Barnabas , Q. E. Bhorat , S. Bibi , C. Briner , P. Cicconi , A. M. Collins , R. Colin‐Jones , C. L. Cutland , T. C. Darton , K. Dheda , C. J. A. Duncan , K. R. W. Emary , K. J. Ewer , L. Fairlie , S. N. Faust , S. Feng , D. M. Ferreira , A. Finn , A. L. Goodman , C. M. Green , C. A. Green , P. T. Heath , et al., South Africa, and the UK 2021, 397, 99.

[4] R. E. Chen , X. Zhang , J. B. Case , E. S. Winkler , Y. Liu , L. A. Vanblargan , J. Liu , J. M. Errico , X. Xie , N. Suryadevara , P. Gilchuk , S. J. Zost , S. Tahan , L. Droit , J. S. Turner , W. Kim , A. J. Schmitz , M. Thapa , D. Wang , A. C. M. Boon , R. M. Presti , J. A. O'halloran , A. H. J. Kim , P. Deepak , D. Pinto , D. H. Fremont , J. E. Crowe , D. Corti , H. W. Virgin , A. H. Ellebedy , et al., Nat. Med. 2021, 27, 717. 33664494 10.1038/s41591-021-01294-w PMC8058618

[5] D. Planas , N. Saunders , P. Maes , F. Guivel‐Benhassine , C. Planchais , J. Buchrieser , W.‐H. Bolland , F. Porrot , I. Staropoli , F. Lemoine , H. Péré , D. Veyer , J. Puech , J. Rodary , G. Baele , S. Dellicour , J. Raymenants , S. Gorissen , C. Geenen , B. Vanmechelen , T. Wawina‐Bokalanga , J. Martí‐Carreras , L. Cuypers , A. Sève , L. Hocqueloux , T. Prazuck , F. A. Rey , E. Simon‐Loriere , T. Bruel , H. Mouquet , et al., Nature 2022, 602, 671. 35016199 10.1038/s41586-021-04389-z

[6] P. Wang , M. S. Nair , L. Liu , S. Iketani , Y. Luo , Y. Guo , M. Wang , J. Yu , B. Zhang , P. D. Kwong , B. S. Graham , J. R. Mascola , J. Y. Chang , M. T. Yin , M. Sobieszczyk , C. A. Kyratsous , L. Shapiro , Z. Sheng , Y. Huang , D. D. Ho , Nature 2021, 593, 130. 33684923 10.1038/s41586-021-03398-2

[7] J. Hansen , A. Baum , K. E. Pascal , V. Russo , S. Giordano , E. Wloga , B. O. Fulton , Y. Yan , K. Koon , K. Patel , K. M. Chung , A. Hermann , E. Ullman , J. Cruz , A. Rafique , T. Huang , J. Fairhurst , C. Libertiny , M. Malbec , W.‐Y. Lee , R. Welsh , G. Farr , S. Pennington , D. Deshpande , J. Cheng , A. Watty , P. Bouffard , R. Babb , N. Levenkova , C. Chen , et al., Science 2020, 369, 1010. 32540901 10.1126/science.abd0827 PMC7299284

[8] A. O. Hassan , J. B. Case , E. S. Winkler , L. B. Thackray , N. M. Kafai , A. L. Bailey , B. T. Mccune , J. M. Fox , R. E. Chen , W. B. Alsoussi , J. S. Turner , A. J. Schmitz , T. Lei , S. Shrihari , S. P. Keeler , D. H. Fremont , S. Greco , P. B. Mccray , S. Perlman , M. J. Holtzman , A. H. Ellebedy , M. S. Diamond , Cell 2020, 182, 744. 32553273 10.1016/j.cell.2020.06.011 PMC7284254

[9] T. F. Rogers , F. Zhao , D. Huang , N. Beutler , A. Burns , W.‐T. He , O. Limbo , C. Smith , G. Song , J. Woehl , L. Yang , R. K. Abbott , S. Callaghan , E. Garcia , J. Hurtado , M. Parren , L. Peng , S. Ramirez , J. Ricketts , M. J. Ricciardi , S. A. Rawlings , N. C. Wu , M. Yuan , D. M. Smith , D. Nemazee , J. R. Teijaro , J. E. Voss , I. A. Wilson , R. Andrabi , B. Briney , et al., Science 2020, 369, 956. 32540903 10.1126/science.abc7520 PMC7299280

[10] A. L. Cathcart , C. Havenar‐Daughton , F. A. Lempp , D. Ma , M. Schmid , M. L. Agostini , B. Guarino , J. Di iulio , L. Rosen , H. Tucker , J. Dillen , S. Subramanian , B. Sloan , S. Bianchi , J. Wojcechowskyj , J. Zhou , H. Kaiser , A. Chase , M. Montiel‐Ruiz , E. Dellota Jr , A. Park , R. Spreafico , A. Sahakyan , E. J. Lauron , N. Czudnochowski , E. Cameroni , S. Ledoux , Y. Kawaoka , A. Werts , et al., bioRxiv 2021, 10.1101/2021.03.09.434607.

[11] D. M. Weinreich , S. Sivapalasingam , T. Norton , S. Ali , H. Gao , R. Bhore , B. J. Musser , Y. Soo , D. Rofail , J. Im , C. Perry , C. Pan , R. Hosain , A. Mahmood , J. D. Davis , K. C. Turner , A. T. Hooper , J. D. Hamilton , A. Baum , C. A. Kyratsous , Y. Kim , A. Cook , W. Kampman , A. Kohli , Y. Sachdeva , X. Graber , B. Kowal , T. Dicioccio , N. Stahl , L. Lipsich , et al., N. Engl. J. Med. 2021, 384, 238. 33332778 10.1056/NEJMoa2035002 PMC7781102

[12] K. Westendorf , S. Žentelis , L. Wang , D. Foster , P. Vaillancourt , M. Wiggin , E. Lovett , R. van der Lee , J. Hendle , A. Pustilnik , J. M. Sauder , L. Kraft , Y. Hwang , R. W. Siegel , J. Chen , B. A. Heinz , R. E. Higgs , N. L. Kallewaard , K. Jepson , R. Goya , M. A. Smith , D. W. Collins , D. Pellacani , P. Xiang , V. de Puyraimond , M. Ricicova , L. Devorkin , C. Pritchard , A. O'Neill , K. Dalal , et al., Cell Rep. 2022, 39, 110812. 35568025 10.1016/j.celrep.2022.110812 PMC9035363

[13] J. Lan , J. Ge , J. Yu , S. Shan , H. Zhou , S. Fan , Q. Zhang , X. Shi , Q. Wang , L. Zhang , X. Wang , Nature 2020, 581, 215. 32225176 10.1038/s41586-020-2180-5

[14] A. C. Walls , Y.‐J. Park , M. A. Tortorici , A. Wall , A. T. Mcguire , D. Veesler , Cell 2020, 183, 1735. 33306958 10.1016/j.cell.2020.11.032 PMC7833104

[15] R. Yan , Y. Zhang , Y. Li , Lu Xia , Y. Guo , Q. Zhou , Science 2020, 367, 1444. 32132184 10.1126/science.abb2762 PMC7164635

[16] C. O. Barnes , C. A. Jette , M. E. Abernathy , K.‐M. A. Dam , S. R. Esswein , H. B. Gristick , A. G. Malyutin , N. G. Sharaf , K. E. Huey‐Tubman , Y. E. Lee , D. F. Robbiani , M. C. Nussenzweig , A. P. West , P. J. Bjorkman , Nature 2020, 588, 682. 33045718 10.1038/s41586-020-2852-1 PMC8092461

[17] M. A. Tortorici , M. Beltramello , F. A. Lempp , D. Pinto , Ha. V. Dang , L. E. Rosen , M. Mccallum , J. Bowen , A. Minola , S. Jaconi , F. Zatta , A. De Marco , B. Guarino , S. Bianchi , E. J. Lauron , H. Tucker , J. Zhou , A. Peter , C. Havenar‐Daughton , J. A. Wojcechowskyj , J. B. Case , R. E. Chen , H. Kaiser , M. Montiel‐Ruiz , M. Meury , N. Czudnochowski , R. Spreafico , J. Dillen , C. Ng , N. Sprugasci , et al., Science 2020, 370, 950. 32972994 10.1126/science.abe3354 PMC7857395

[18] C. Liu , H. M. Ginn , W. Dejnirattisai , P. Supasa , B. Wang , A. Tuekprakhon , R. Nutalai , D. Zhou , A. J. Mentzer , Y. Zhao , H. M. E. Duyvesteyn , C. López‐Camacho , J. Slon‐Campos , T. S. Walter , D. Skelly , S. A. Johnson , T. G. Ritter , C. Mason , S. A. Costa Clemens , F. Gomes Naveca , V. Nascimento , F. Nascimento , C. Fernandes Da Costa , P. C. Resende , A. Pauvolid‐Correa , M. M. Siqueira , C. Dold , N. Temperton , T. Dong , A. J. Pollard , et al., Cell 2021, 184, 4220. 34242578 10.1016/j.cell.2021.06.020 PMC8218332

[19] P. Supasa , D. Zhou , W. Dejnirattisai , C. Liu , A. J. Mentzer , H. M. Ginn , Y. Zhao , H. M. E. Duyvesteyn , R. Nutalai , A. Tuekprakhon , B. Wang , G. C. Paesen , J. Slon‐Campos , C. López‐Camacho , B. Hallis , N. Coombes , K. R. Bewley , S. Charlton , T. S. Walter , E. Barnes , S. J. Dunachie , D. Skelly , S. F. Lumley , N. Baker , I. Shaik , H. E. Humphries , K. Godwin , N. Gent , A. Sienkiewicz , C. Dold , et al., Cell 2021, 184, 2201. 33743891 10.1016/j.cell.2021.02.033 PMC7891044

[20] S. Muyldermans , Annu. Rev. Biochem. 2013, 82, 775. 23495938 10.1146/annurev-biochem-063011-092449

[21] M. Scully , S. R. Cataland , F. Peyvandi , P. Coppo , P. Knöbl , J. A. Kremer Hovinga , A. Metjian , J. De La Rubia , K. Pavenski , F. Callewaert , D. Biswas , H. De Winter , R. K. Zeldin , N. Engl. J. Med. 2019, 380, 335.30625070

[22] J. Huo , A. Le Bas , R. R. Ruza , H. M. E. Duyvesteyn , H. Mikolajek , T. Malinauskas , T. K. Tan , P. Rijal , M. Dumoux , P. N. Ward , J. Ren , D. Zhou , P. J. Harrison , M. Weckener , D. K. Clare , V. K. Vogirala , J. Radecke , L. Moynié , Y. Zhao , J. Gilbert‐Jaramillo , M. L. Knight , J. A. Tree , K. R. Buttigieg , N. Coombes , M. J. Elmore , M. W. Carroll , L. Carrique , P. N. M. Shah , W. James , A. R. Townsend , et al., Nat. Struct. Mol. Biol. 2020, 27, 846. 32661423 10.1038/s41594-020-0469-6

[23] P.‐A. Koenig , H. Das , H. Liu , B. M. Kümmerer , F. N. Gohr , L.‐M. Jenster , L. D. J. Schiffelers , Y. M. Tesfamariam , M. Uchima , J. D. Wuerth , K. Gatterdam , N. Ruetalo , M. H. Christensen , C. I. Fandrey , S. Normann , J. M. P. Tödtmann , S. Pritzl , L. Hanke , J. Boos , M. Yuan , X. Zhu , J. L. Schmid‐Burgk , H. Kato , M. Schindler , I. A. Wilson , M. Geyer , K. U. Ludwig , B. M Hällberg , N. C. Wu , F. I. Schmidt , Science 2021, 371, eabe6230. 33436526 10.1126/science.abe6230 PMC7932109

[24] D. Wrapp , D. De Vlieger , K. S. Corbett , G. M. Torres , N. Wang , W. Van Breedam , K. Roose , L. Van Schie , M. Hoffmann , S. Pöhlmann , B. S. Graham , N. Callewaert , B. Schepens , X. Saelens , J. S. Mclellan , Cell 2020, 181, 1004. 32375025 10.1016/j.cell.2020.04.031 PMC7199733

[25] J. Xu , K. Xu , S. Jung , A. Conte , J. Lieberman , F. Muecksch , J. C. C. Lorenzi , S. Park , F. Schmidt , Z. Wang , Y. Huang , Y. Luo , M. S. Nair , P. Wang , J. E. Schulz , L. Tessarollo , T. Bylund , G‐Yu Chuang , A. S. Olia , T. Stephens , I.‐T. Teng , Y. Tsybovsky , T. Zhou , V. Munster , D. D. Ho , T. Hatziioannou , P. D. Bieniasz , M. C. Nussenzweig , P. D. Kwong , R. Casellas , Nature 2021, 595, 278. 34098567 10.1038/s41586-021-03676-z PMC8260353

[26] J. Huo , H. Mikolajek , A. Le Bas , J. J. Clark , P. Sharma , A. Kipar , J. Dormon , C. Norman , M. Weckener , D. K. Clare , P. J. Harrison , J. A. Tree , K. R. Buttigieg , F. J. Salguero , R. Watson , D. Knott , O. Carnell , D. Ngabo , M. J. Elmore , S. Fotheringham , A. Harding , L. Moynie? , P. N. Ward , M. Dumoux , T. Prince , Y. Hall , J. A. Hiscox , A. Owen , W. James , M. W. Carroll , et al., Nat. Commun. 2021, 12, 5469. 34552091 10.1038/s41467-021-25480-z PMC8458290

[27] S. Nambulli , Y. Xiang , N. L. Tilston‐Lunel , L. J. Rennick , Z. Sang , W. B. Klimstra , D. S. Reed , N. A. Crossland , Y. Shi , W. P. Duprex , Sci. Adv. 2021, 7, eabh0319. 34039613 10.1126/sciadv.abh0319 PMC8153718

[28] P. Pymm , A. Adair , L.‐J. Chan , J. P. Cooney , F. L. Mordant , C. C. Allison , E. Lopez , E. R. Haycroft , M. T. O'neill , Li. L Tan , M. H. Dietrich , D. Drew , M. Doerflinger , M. A. Dengler , N. E. Scott , A. K. Wheatley , N. A. Gherardin , H. Venugopal , D. Cromer , M. P. Davenport , R. Pickering , D. I. Godfrey , D. F. J. Purcell , S. J. Kent , A. W. Chung , K. Subbarao , M. Pellegrini , A. Glukhova , W.‐H. Tham , Proc. Natl. Acad. Sci. USA 2021, 118, 12. 10.1073/pnas.2101918118 PMC8126837 33893175

[29] J. D. Walter , M. Scherer , C. A. J. Hutter , A. A. Garaeva , I. Zimmermann , M. Wyss , J. Rheinberger , Y. Ruedin , J. C. Earp , P. Egloff , M. Sorgenfrei , L. M. Hürlimann , I. Gonda , G. Meier , S. Remm , S. Thavarasah , G. Van Geest , R. Bruggmann , G. Zimmer , D. J. Slotboom , C. Paulino , P. Plattet , M. A. Seeger , EMBO Rep. 2022, 23, e54199. 35253970 10.15252/embr.202154199 PMC8982573

[30] Y. Xiang , S. Nambulli , Z. Xiao , H. Liu , Z. Sang , W. P Duprex , D. Schneidman‐Duhovny , C. Zhang , Y. Shi , Science 2020, 370, 1479. 33154108 10.1126/science.abe4747 PMC7857400

[31] T. Güttler , M. Aksu , A. Dickmanns , K. M. Stegmann , K. Gregor , R. Rees , W. Taxer , O. Rymarenko , J. Schünemann , C. Dienemann , P. Gunkel , B. Mussil , J. Krull , U. Teichmann , U. Groß , V. C. Cordes , M. Dobbelstein , D. Görlich , EMBO J. 2021, 40, 107985. 10.15252/embj.2021107985 PMC8420576 34302370

[32] E. Segura , J. A. Villadangos , Curr. Opin. Immunol. 2009, 21, 105. 19342210 10.1016/j.coi.2009.03.011

[33] I. Caminschi , M. H. Lahoud , K. Shortman , Eur. J. Immunol. 2009, 39, 931. 19197943 10.1002/eji.200839035

[34] Y. Do , H. Koh , C. G. Park , D. Dudziak , P. Seo , S. Mehandru , J.‐H. Choi , C. Cheong , S. Park , D. S. Perlin , B. S. Powell , R. M. Steinman , Eur. J. Immunol. 2010, 40, 2791. 20812236 10.1002/eji.201040511

[35] C. Napoletano , A. Rughetti , M. P. Agervig Tarp , J. Coleman , E. P. Bennett , G. Picco , P. Sale , K. Denda‐Nagai , T. Irimura , U. Mandel , H. Clausen , L. Frati , J. Taylor‐Papadimitriou , J. Burchell , M. Nuti , Cancer Res. 2007, 67, 8358. 17804752 10.1158/0008-5472.CAN-07-1035

[36] B. Wang , N. Zaidi , Li‐Z He , Li Zhang , J. My Kuroiwa , T. Keler , R. M. Steinman , Breast Cancer Res. 2012, 14, R39. 22397502 10.1186/bcr3135 PMC3446373

[37] D. Sancho , D. Mourão‐Sá , O. P. Joffre , O. Schulz , N. C. Rogers , D. J. Pennington , J. R. Carlyle , C. Reis E Sousa , J. Clin. Invest. 2008, 118, 2098. 18497879 10.1172/JCI34584 PMC2391066

[38] L. F. Poulin , M. Salio , E. Griessinger , F. Anjos‐Afonso , L. Craciun , Ji‐Li Chen , A. M. Keller , O. Joffre , S. Zelenay , E. Nye , A. Le Moine , F. Faure , V. Donckier , D. Sancho , V. Cerundolo , D. Bonnet , C. Reis E Sousa , J. Exp. Med. 2010, 207, 1261. 20479117 10.1084/jem.20092618 PMC2882845

[39] C. Huysamen , J. A. Willment , K. M. Dennehy , G. D. Brown , J. Biol. Chem. 2008, 283, 16693. 18408006 10.1074/jbc.M709923200 PMC2562446

[40] D. Sancho , O. P. Joffre , A. M. Keller , N. C. Rogers , D. Martínez , P. Hernanz‐Falcón , I. Rosewell , C. R. E. Sousa , Nature 2009, 458, 899. 19219027 10.1038/nature07750 PMC2671489

[41] S. Zelenay , A. M. Keller , P. G. Whitney , B. U. Schraml , S. Deddouche , N. C. Rogers , O. Schulz , D. Sancho , C. Reis E Sousa , J. Clin. Invest. 2012, 122, 1615. 22505458 10.1172/JCI60644 PMC3336984

[42] S. Iborra , M. Martínez‐López , S. C. Khouili , M. Enamorado , F. J. Cueto , R. Conde‐Garrosa , C. Del Fresno , D. Sancho , Immunity 2016, 45, 847. 27692611 10.1016/j.immuni.2016.08.019 PMC5074364

[43] A. Blanco‐Toribio , N. Sainz‐Pastor , A. Álvarez‐Cienfuegos , N. Merino , Á. M. Cuesta , D. Sánchez‐Martín , J. Bonet , P. Santos‐Valle , L. Sanz , B. Oliva , F. J. Blanco , L. Álvarez‐Vallina , mAbs 2013, 5, 70. 23221741 10.4161/mabs.22698 PMC3564888

[44] M. Compte , S. L. Harwood , A. Erce‐Llamazares , A. Tapia‐Galisteo , E. Romero , I. Ferrer , E. M. Garrido‐Martin , A. B. Enguita , M. C. Ochoa , B. Blanco , M. Oteo , N. Merino , D. Nehme‐Álvarez , O. Hangiu , C. Domínguez‐Alonso , M. Zonca , A. Ramírez‐Fernández , F. J. Blanco , M. A. Morcillo , I. G. Muñoz , I. Melero , J. L. Rodriguez‐Peralto , L. Paz‐Ares , L. Sanz , L. Alvarez‐Vallina , Clin. Cancer Res. 2021, 27, 3167. 33785484 10.1158/1078-0432.CCR-20-4625

[45] G. B. Moreau , S. L. Burgess , J. M. Sturek , A. N. Donlan , W. A. Petri , B. J. Mann , Am. J. Trop. Med. Hyg. 2020, 103, 1215. 32723427 10.4269/ajtmh.20-0762 PMC7470527

[46] W. Du , P. Jiang , Q. Li , H. Wen , M. Zheng , J. Zhang , Y. Guo , J. Yang , W. Feng , S. Ye , S. Kamara , P. Jiang , J. Chen , W. Li , S. Zhu , L. Zhang , Microbiol. Spectr. 2023, 11, e0356222. 36511681 10.1128/spectrum.03562-22 PMC9927262

[47] D. Li , G. D. Sempowski , K. O. Saunders , P. Acharya , B. F. Haynes , Annu. Rev. Med. 2022, 73, 1. 34428080 10.1146/annurev-med-042420-113838

[48] E. M. Obeng , C. K. O. Dzuvor , M. K. Danquah , Nano Today 2022, 42, 101350. 34840592 10.1016/j.nantod.2021.101350 PMC8608585

[49] N. Suryadevara , S. Shrihari , P. Gilchuk , L. A. Vanblargan , E. Binshtein , S. J. Zost , R. S. Nargi , R. E. Sutton , E. S. Winkler , E. C. Chen , M. E. Fouch , E. Davidson , B. J. Doranz , R. E. Chen , P.‐Y. Shi , R. H. Carnahan , L. B. Thackray , M. S. Diamond , J. E. Crowe , Cell 2021, 184, 2316. 33773105 10.1016/j.cell.2021.03.029 PMC7962591

[50] I. Ullah , J. Prévost , M. S. Ladinsky , H. Stone , M. Lu , S. P. Anand , G. Beaudoin‐Bussières , K. Symmes , M. Benlarbi , S. Ding , R. Gasser , C. Fink , Y. Chen , A. Tauzin , G. Goyette , C. Bourassa , H. Medjahed , M. Mack , K. Chung , C. B. Wilen , G. A. Dekaban , J. D. Dikeakos , E. A. Bruce , D. E. Kaufmann , L. Stamatatos , A. T. McGuire , J. Richard , M. Pazgier , P. J. Bjorkman , W. Mothes , et al., bioRxiv 2021, 10.1101/2021.03.22.436337.

[51] E. S. Winkler , P. Gilchuk , J. Yu , A. L. Bailey , R. E. Chen , Z. Chong , S. J. Zost , H. Jang , Y. Huang , J. D. Allen , J. B. Case , R. E. Sutton , R. H. Carnahan , T. L. Darling , A. C. M. Boon , M. Mack , R. D. Head , T. M. Ross , J. E. Crowe , M. S. Diamond , Cell 2021, 184, 1804. 33691139 10.1016/j.cell.2021.02.026 PMC7879018

[52] R. Yamin , A. T. Jones , H.‐H. Hoffmann , A. Schäfer , K. S. Kao , R. L. Francis , T. P. Sheahan , R. S. Baric , C. M. Rice , J. V. Ravetch , S. Bournazos , Nature 2021, 599, 465. 34547765 10.1038/s41586-021-04017-w PMC9038156

[53] Y. Wang , Y. Xiang , V. W. Xin , X.‐W. Wang , X.‐C. Peng , X.‐Q. Liu , D. Wang , N. Li , J.‐T. Cheng , Y.‐N. Lyv , S.‐Z. Cui , Z. Ma , Q. Zhang , H.‐W. Xin , J. Hematol. Oncol. 2020, 13, 107. 32746880 10.1186/s13045-020-00939-6 PMC7397618

[54] S. He , J. Wang , H. Chen , Z. Qian , K. Hu , B. Shi , J. Wang , Vaccines 2023, 11, 371. 36851249 10.3390/vaccines11020371 PMC9964001

[55] C. Li , W. Zhan , Z. Yang , C. Tu , G. Hu , X. Zhang , W. Song , S. Du , Y. Zhu , K. Huang , Yu Kong , M. Zhang , Q. Mao , X. Gu , Yi Zhang , Y. Xie , Q. Deng , Y. Song , Z. Chen , L. Lu , S. Jiang , Y. Wu , L. Sun , T. Ying , Cell 2022, 185, 1389. 35344711 10.1016/j.cell.2022.03.009 PMC8907017

[56] M. A. Rossotti , H. Van Faassen , A. T. Tran , J. Sheff , J. K. Sandhu , D. Duque , M. Hewitt , X. Wen , J. Bavananthasivam , S. Beitari , K. Matte , G. Laroche , P. M. Giguère , C. Gervais , M. Stuible , J. Guimond , S. Perret , G. Hussack , M.‐A. Langlois , Y. Durocher , J. Tanha , Commun. Biol. 2022, 5, 933. 36085335 10.1038/s42003-022-03866-z PMC9461429

[57] A. Gupta , Y. Gonzalez‐Rojas , E. Juarez , M. Crespo Casal , J. Moya , D. R. Falci , E. Sarkis , J. Solis , H. Zheng , N. Scott , A. L. Cathcart , C. M. Hebner , J. Sager , E. Mogalian , C. Tipple , A. Peppercorn , E. Alexander , P. S. Pang , A. Free , C. Brinson , M. Aldinger , A. E. Shapiro , N. Engl. J. Med. 2021, 385, 1941. 34706189 10.1056/NEJMoa2107934

[58] J. J. Guthmiller , J. Han , H. A. Utset , L. Li , L. Y.‐L Lan , C. Henry , C. T. Stamper , M. McMahon , G. O'dell , M. L. Fernández‐Quintero , A. W. Freyn , F. Amanat , O. Stovicek , L. Gentles , S. T. Richey , A. T. De La Peña , V. Rosado , H. L. Dugan , N.‐Y. Zheng , M. E. Tepora , D. J. Bitar , S. Changrob , S. Strohmeier , M. Huang , A. García‐Sastre , K. R. Liedl , J. D. Bloom , R. Nachbagauer , P. Palese , F. Krammer , et al., Nature 2022, 602, 314. 34942633 10.1038/s41586-021-04356-8 PMC8828479

[59] D. Sok , D. R. Burton , Nat. Immunol. 2018, 19, 1179. 30333615 10.1038/s41590-018-0235-7 PMC6440471

[60] A. Tang , Z. Chen , K. S. Cox , H.‐P. Su , C. Callahan , A. Fridman , L. Zhang , S. B. Patel , P. J. Cejas , R. Swoyer , S. Touch , M. P. Citron , D. Govindarajan , B. Luo , M. Eddins , J. C. Reid , S. M. Soisson , J. Galli , D. Wang , Z. Wen , G. J. Heidecker , D. R. Casimiro , D. J. Distefano , K. A. Vora , Nat. Commun. 2019, 10, 4153. 31515478 10.1038/s41467-019-12137-1 PMC6742648

[61] A. R. Mäkelä , H. Ugurlu , L. Hannula , R. Kant , P. Salminen , R. Fagerlund , S. Mäki , A. Haveri , T. Strandin , L. Kareinen , J. Hepojoki , S. Kuivanen , L. Levanov , A. Pasternack , R. A. Naves , O. Ritvos , P. Österlund , T. Sironen , O. Vapalahti , A. Kipar , J. T. Huiskonen , I. Rissanen , K. Saksela , Nat. Commun. 2023, 14, 1637. 36964125 10.1038/s41467-023-37290-6 PMC10037368

[62] G. Beaudoin‐Bussières , Y. Chen , I. Ullah , J. Prévost , W. D. Tolbert , K. Symmes , S. Ding , M. Benlarbi , S. Yu Gong , A. Tauzin , R. Gasser , D. Chatterjee , D. Vézina , G. Goyette , J. Richard , F. Zhou , L. Stamatatos , A. T. Mcguire , H. Charest , M. Roger , E. Pozharski , P. Kumar , W. Mothes , P. D. Uchil , M. Pazgier , A. Finzi , Cell Rep. 2022, 38, 110368. 35123652 10.1016/j.celrep.2022.110368 PMC8786652

[63] C. Kurts , W. R. Heath , F. R. Carbone , J. Allison , J. F. Miller , H. Kosaka , J. Exp. Med. 1996, 184, 923. 9064352 10.1084/jem.184.3.923 PMC2192761

[64] L. F. Poulin , Y. Reyal , H. Uronen‐Hansson , B. U. Schraml , D. Sancho , K. M. Murphy , U. K. Håkansson , L. Ferreira Moita , W. W. Agace , D. Bonnet , C. Reis E Sousa , Blood 2012, 119, 6052. 22442345 10.1182/blood-2012-01-406967

[65] S. A. Fuertes Marraco , F. Grosjean , A. Duval , M. Rosa , C. Lavanchy , D. Ashok , S. Haller , L. A. Otten , Q.‐G. Steiner , P. Descombes , C. A. Luber , F. Meissner , M. Mann , L. Szeles , W. Reith , H. Acha‐Orbea , Front. Immunol. 2012, 3, 331. 23162549 10.3389/fimmu.2012.00331 PMC3491238

[66] J. M. Díez , C. Romero , J. Vergara‐Alert , M. Belló‐Perez , J. Rodon , J. M. Honrubia , J. Segalés , I. Sola , L. Enjuanes , R. Gajardo , Immunotherapy 2020, 12, 1247. 32900263 10.2217/imt-2020-0220 PMC7480323

[67] P. Pérez , A. Lázaro‐Frías , C. Zamora , P. J. Sánchez‐Cordón , D. Astorgano , J. Luczkowiak , R. Delgado , J. M. Casasnovas , M. Esteban , J. García‐Arriaza , Front. Immunol. 2022, 12, 824728. 35154086 10.3389/fimmu.2021.824728 PMC8829548

[68] C. Hsieh , J. A. Goldsmith , J. M. Schaub , A. M. Divenere , H. Kuo , K. Javanmardi , K. C. Le , D. Wrapp , A. G. Lee , Y. Liu , C. Chou , P. O. Byrne , C. K. Hjorth , N. V Johnson , J. Ludes‐meyers , A. W. Nguyen , J. Park , N. Wang , D. Amengor , J. J. Lavinder , G. C. Ippolito , J. A. Maynard , I. J. Finkelstein , J. S. Mclellan , Science 2020, 369, 1501. 32703906 10.1126/science.abd0826 PMC7402631

[69] Y. Zhang , E. V. Yates , L. Hong , K. L. Saar , G. Meisl , C. M. Dobson , T. P. J. Knowles , Chem. Sci. 2018, 9, 3503. 29780480 10.1039/c7sc04331g PMC5934698

[70] D. K. Wilkins , S. B. Grimshaw , V. Receveur , C. M. Dobson , J. A. Jones , L. J. Smith , Biochemistry 1999, 38, 16424. 10600103 10.1021/bi991765q

[71] J. M. Schaub , C.‐W. Chou , H.‐C. Kuo , K. Javanmardi , C.‐L. Hsieh , J. Goldsmith , A. M. Divenere , K. C. Le , D. Wrapp , P. O. Byrne , C. K. Hjorth , N. V. Johnson , J. Ludes‐Meyers , A. W. Nguyen , N. Wang , J. J. Lavinder , G. C. Ippolito , J. A. Maynard , J. S. Mclellan , I. J. Finkelstein , Nat. Protoc. 2021, 16, 5339. 34611365 10.1038/s41596-021-00623-0 PMC9665560

[72] A. Punjani , J. L. Rubinstein , D. J. Fleet , M. A. Brubaker , Nat. Methods 2017, 14, 290. 28165473 10.1038/nmeth.4169

[73] P. V. Afonine , B. K. Poon , R. J. Read , O. V. Sobolev , T. C. Terwilliger , A. Urzhumtsev , P. D. Adams , Acta Crystallogr., Sect. D: Struct. Biol. 2018, 74, 531. 29872004 10.1107/S2059798318006551 PMC6096492

[74] P. Emsley , K. Cowtan , Acta Crystallogr. D: Biol. Crystallogr. 2004, 60, 2126. 15572765 10.1107/S0907444904019158

[75] E. F. Pettersen , T. D. Goddard , C. C. Huang , E. C. Meng , G. S. Couch , T. I. Croll , J. H. Morris , T. E. Ferrin , Protein Sci. 2021, 30, 70. 32881101 10.1002/pro.3943 PMC7737788

[76] P. D. Adams , P. V. Afonine , G. Bunkóczi , V. B. Chen , I. W. Davis , N. Echols , J. J. Headd , Li‐W Hung , G. J. Kapral , R. W. Grosse‐Kunstleve , A. J. Mccoy , N. W. Moriarty , R. Oeffner , R. J. Read , D. C. Richardson , J. S. Richardson , T. C. Terwilliger , P. H. Zwart , Acta Crystallogr. D: Biol. Crystallogr. 2010, 66, 213. 20124702 10.1107/S0907444909052925 PMC2815670

[77] M. A. Whitt , J. Virol. Methods 2010, 169, 365. 20709108 10.1016/j.jviromet.2010.08.006 PMC2956192

[78] A. Lázaro‐Frías , P. Pérez , C. Zamora , P. J. Sánchez‐Cordón , M. Guzmán , J. Luczkowiak , R. Delgado , J. M. Casasnovas , M. Esteban , J. García‐Arriaza , NPJ Vaccines 2022, 7, 17. 35140227 10.1038/s41541-022-00440-w PMC8828760

[79] J. García‐Arriaza , U. Garaigorta , P. Pérez , A. Lázaro‐Frías , C. Zamora , P. Gastaminza , C. Del Fresno , J. M. Casasnovas , C. Ó. S. Sorzano , D. Sancho , M. Esteban , J. Virol. 2021, 95, e02260. 33414159 10.1128/JVI.02260-20 PMC8092708

[80] H. Hartman , Y. Wang , H. W. Schroeder , X. Cui , PLoS One 2018, 13, e0198528. 29883460 10.1371/journal.pone.0198528 PMC5993274

