# Supplementary material for: Dendritic Cell‐Mediated Cross‐Priming by a Bispecific Neutralizing Antibody Boosts Cytotoxic T Cell Responses and Protects Mice against SARS‐CoV‐2
Source: Adv Sci (Weinh). 2023 Oct 20;10(34):2304818. doi: 10.1002/advs.202304818 (PMC10700188; doi:10.1002/advs.202304818)
Supplement: Supplementary file 1 — Supporting Information [file ADVS-10-2304818-s001.pdf]

ADVANCED SCIENCE
Open Access

Supporting Information

for *Adv. Sci.*, DOI 10.1002/advs.202304818

Dendritic Cell-Mediated Cross-Priming by a Bispecific Neutralizing Antibody Boosts Cytotoxic T Cell Responses and Protects Mice against SARS-CoV-2

*Rodrigo Lázaro-Gorines, Patricia Pérez, Ignacio Heras-Murillo, Irene Adán-Barrientos, Guillermo Albericio, David Astorgano, Sara Flores, Joanna Luczkowiak, Nuria Labiod, Seandean L. Harwood, Alejandro Segura-Tudela, Laura Rubio-Pérez, Yudhi Nugraha, Xiaoran Shang, Yuxing Li, Carlos Alfonso, Kaylin A. Adipietro, Dinendra L. Abeyawardhane, Rocío Navarro, Marta Compte, Wenbo Yu, Alexander D. MacKerell Jr, Laura Sanz, David J. Weber, Francisco J. Blanco, Mariano Esteban, Edwin Pozharski, Raquel Godoy-Ruiz, Inés G. Muñoz, Rafael Delgado, David Sancho, Juan García-Arriaza and Luis Álvarez-Vallina**

Supporting Information

Title

Dendritic cell-mediated cross-priming by a bispecific neutralizing antibody boosts cytotoxic T cell responses and protects mice against SARS-CoV-2

*Rodrigo Lázaro-Gorines, Patricia Pérez, Ignacio Heras-Murillo, Irene Adán-Barrientos, Guillermo Albericio, David Astorgano, Sara Flores, Joanna Luczkowiak, Nuria Labiod, Seandean L. Harwood, Alejandro Segura-Tudela, Laura Rubio-Pérez, Yudhi Nugraha, Xiaoran Shang, Yuxing Li, Carlos Alfonso, Kaylin A. Adipietro, Dinendra L. Abeyawardhane, Rocío Navarro, Marta Compte, Wenbo Yu, Alexander D. MacKerell Jr, Laura Sanz, David J. Weber, Francisco J. Blanco, Mariano Esteban, Edwin Pozharski, Raquel Godoy-Ruiz, Inés G. Muñoz, Rafael Delgado, David Sancho, Juan García-Arriaza, Luis Álvarez-Vallina**

*** Correspondence: lalvarezv@ext.cnio.es (L. A-V)**

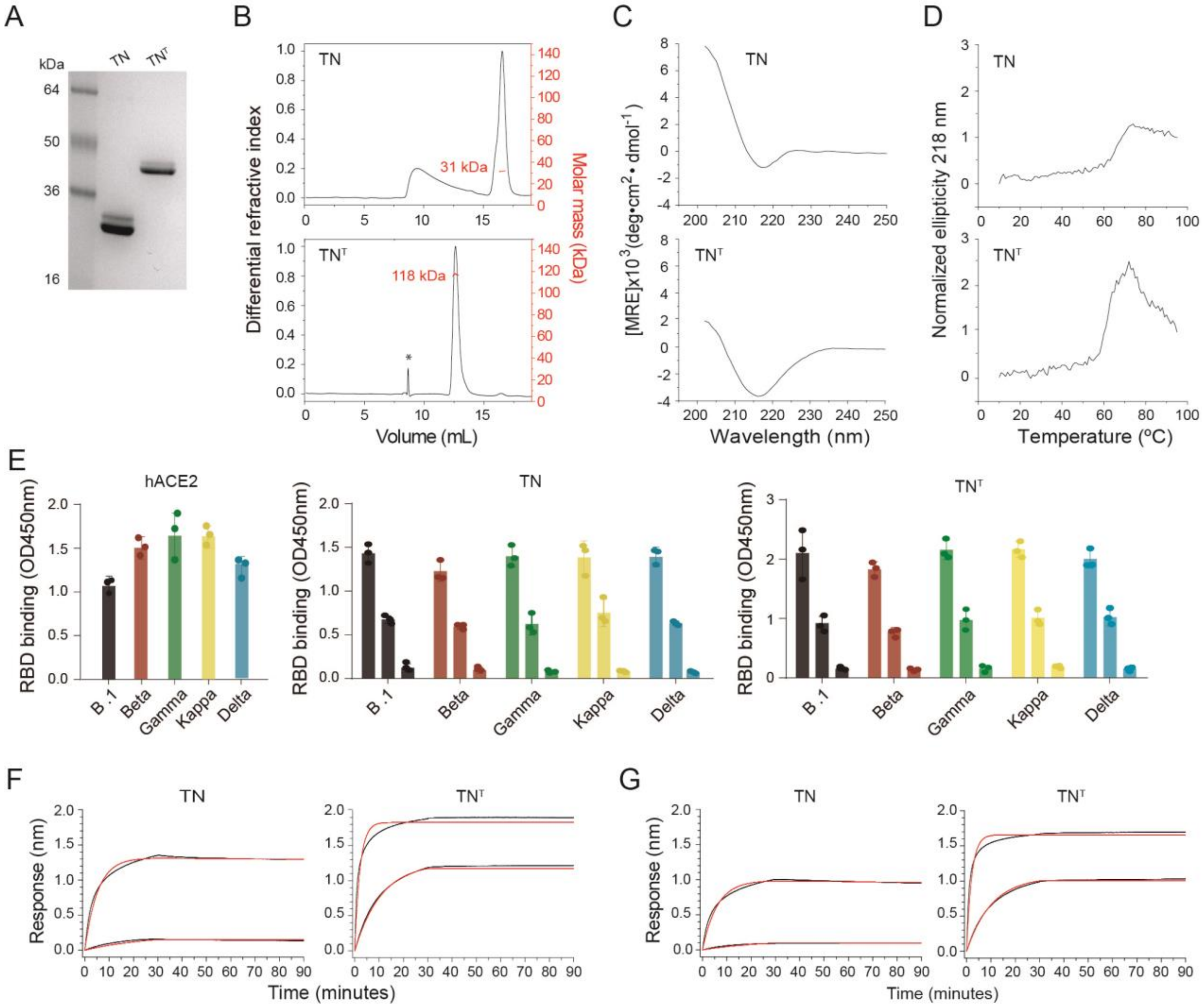

Figure S1. Purified TN tandem and TN^{T} trimerbody recognize efficiently and similarly SARS-CoV-2 S protein RBD from different viral strains. A) SDS-PAGE analysis in reducing conditions of purified TN and TN^{T}. Both antibodies were purified from HEK-293 conditioned medium. B) SEC-MALS data proving monomeric (TN, top) and trimeric (TN^{T}, bottom) states of each antibody. The black lines correspond to the differential refractive index (left axis) and the red line to the measured molar mass (right axis). Asterisk (*) in TN^{T} chromatogram indicates an experimental artifact spike cause by a chromatography pause. C) TN (top) and TN^{T} (bottom) far-UV CD spectra. D) TN (top) and TN^{T} (bottom) thermal denaturation profiles. E) TN (50, 10, 1 nM) and TN^{T} (10, 1, 0.1 nM) binding activity against immobilized B.1, beta, gamma, kappa and delta RBD proteins. ACE2 (0.1 µg/mL) interaction with different RBD strains was included as an inner control (left panel). All assays were performed by triplicate. Independent and mean values ± SEM are plotted. The binding kinetics of the TN and TN^{T} to B.1 (F) and B.1.351(beta) (G) RBD were measured, using the antibodies at 50 and 10 nM, with 0.5

hours of association and 1 hour of dissociation. Experimental sensorgrams (black) and fitted data (red) are shown.

A

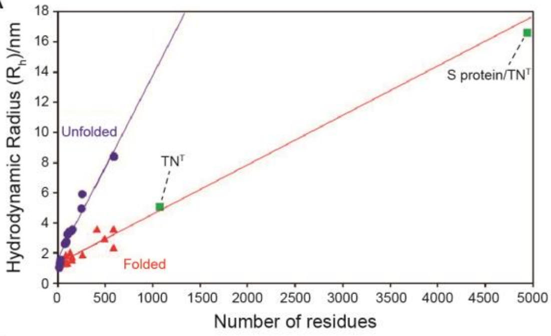

B

Unfolded proteins

Protein	No.of residues	R_h/nm
Hen lysozyme (49-64)	16	1.04
D3 fibronectin binding protein (17-37)	22	1.33
D3 fibronectin binding protein (2-38)	32	1.55
Bovine ubiquitin	76	2.63
SH3 domain of PI3K	90	2.75
Horse ferricytochrome c	104	3.24
Hen lysozyme	129	3.46
Staphylococcal nuclease	149	3.5
Horse myoglobin	153	3.6
Yeast triosephosphate isomerase	247	4.97
Bovine carbonic anhydrase B	259	5.9
BSA	589	8.4

Folded proteins

Protein	No. of residues	R_h/nm
Bovine pancreatic trypsin inhibitor	58	1.58
Bovine ubiquitin	76	1.32
SH3 domain of PI3K	90	1.86
R_h/nmHorse ferricytochrome c	104	1.35
Hen lysozyme	129	2.05
Staphylococcal nuclease	149	1.6
Horse myoglobin	153	1.75
Bovine carbonic anhydrase B	259	1.9
S. equismilis streptokinase	414	3.6
Yeast triosephosphate isomerase	494	2.97
Yeast triosephosphate isomerase	589	2.35
BSA	589	3.6

TN^T and S protein/TN^T

Protein	No. of residues	R_h/nm
TN^T	1077 (359 x 3)	5.06
S protein+TN^T(1:1)	4941 (3864+1077)	16.6

C

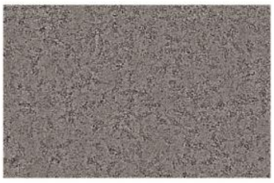

D

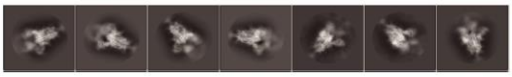

E

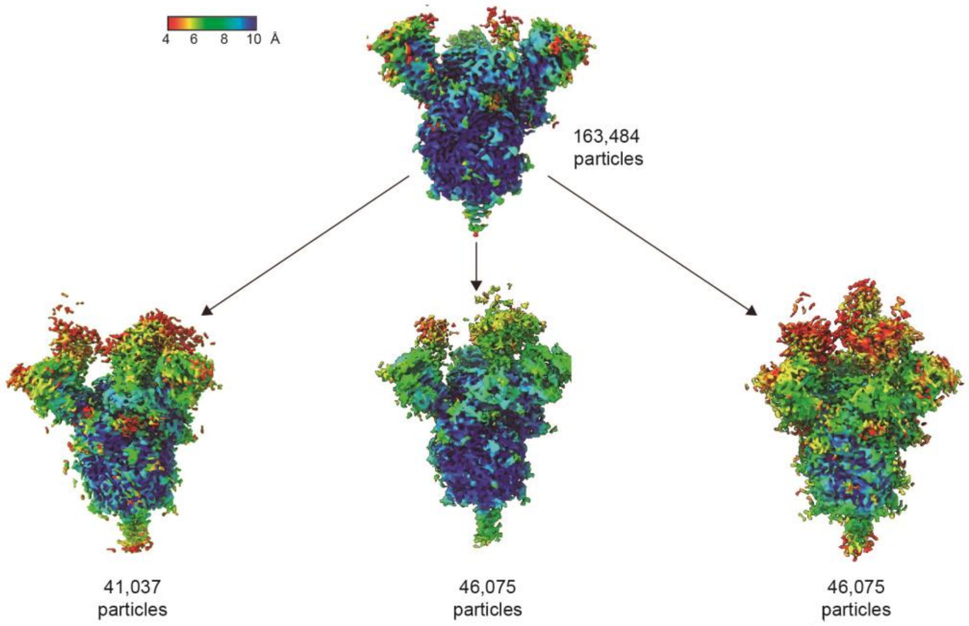

F

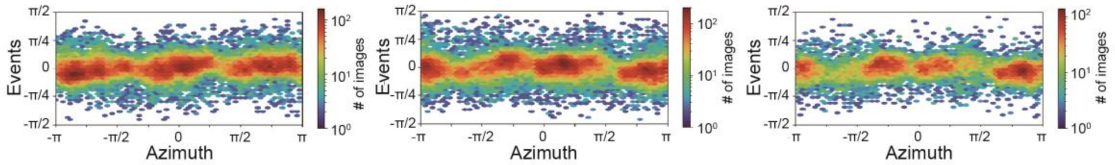

G

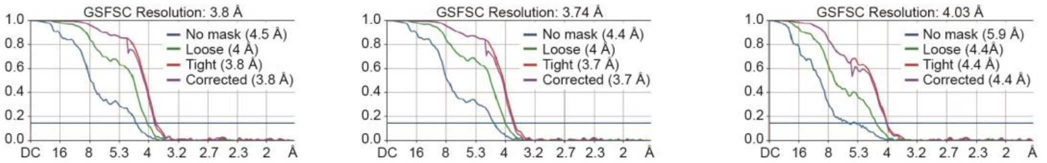

H

Details of cryo-EM data collection

Microscope	FEI Glacios
Voltage (kV)	200
Camera	Gatan K3
Defocus range used (µm)	0.5 – 2.7
Exposure time	3.0
Dose rate (e/pixel/s)	16.3
Total dose (e/Å^2)	61.9
Pixel size (Å)	0.889
Number of micrographs (total/used)	2184/1980
Frames	46
Total Particles Picked	163,484
Particles used per 3D class	22,422/46,075/41,037
Resolution in 3D classes(Å)	3.80/3.74/4.03

Figure S2. Microfluidic diffusional sizing (MDS) analysis and cryo-EM data processing and reconstruction of HexaPro S protein/TN^T complex. MDS was used to measure the change in hydrodynamic radius (R_h) of TN^T trimerbody and its subsequent complex with SARS-CoV-2 S protein. A) Plot of the R_h versus the number of residues in the polypeptide chain. The values of the hydrodynamic radii displayed in this graph correspond to the R_h values reported in the literature for a range of native folded proteins (red) and highly denatured polypeptide chains (blue) obtained using dynamic light scattering and pulsed field gradient nuclear magnetic resonance. TN^T and S protein/TN^T complex R_hs (green) correlate with the expected for folded proteins with their nominal number of amino acids. B) Charts indicating the number of residues and R_h of folded (red) and unfolded (blue) polypeptides and proteins used in this correlation according to literature. Number of residues of TN^T, 1077, and S protein/TN^T complex, 4941, trimeric conformations in solution (green). C-H, cryo-EM data processing and reconstruction of HexaPro S protein/TN^T complex**.** C) Regarding Cryo-EM analyses top panel shows an example of motion corrected micrograph. D) Subset of 7 2D classes obtained from the 2D classification job run after particle picking. E) Electron density maps representative of final 3D complex reconstruction at 3.8Å and local map resolution. Three volume subclasses were identified as the majoritarian ones. F) Viewing Direction Distribution map illustrating how many images have a viewing direction at each (elevation, azimuth) bin. G) Validation and resolution estimation results. Fourier Shell correlation (FSC) curves are measured between half-sets of particles that are used to compute two half-map reconstructions. FSC curves using a mask (green, red, purple) improved the resolution estimate of 3.8Å versus 4.5Å for conventional refinement. Notably, a local focused refinement using the same mask is unable to improve resolution beyond the conventional refinement result due to the flexibility of the region. H) Cryo-EM data collecting and analysis table.

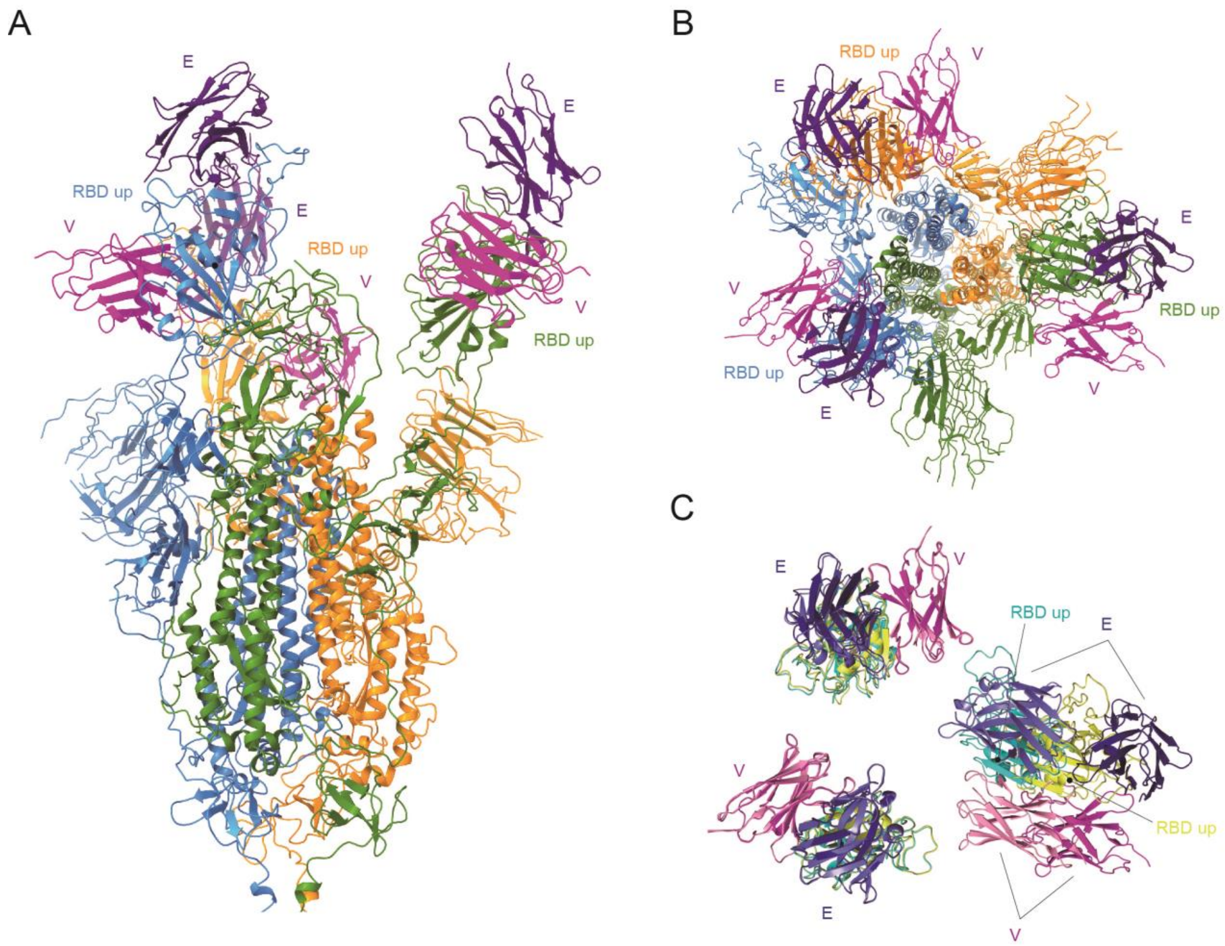

Figure S3. S protein/TN^T complex model shows TN^T hexavalent binding to S protein causing the 3-up RBD conformation with one-RBD position distortion. Side (A) and top views (B) of the HexaPro S protein/TN^T complex model showing TN^T embracing the S protein in the 3-up RBD prefusion conformation. As shown, TN^T binds simultaneously and in a biparatopic manner, both E V_{HH} and V V_{HH}, to the three S protein RBDs. S protein subunits are colored in yellow, steel blue and olive green, while E V_{HH} and V V_{HH} are in purple and magenta, respectively. C) Top view of the alignment of the HexaPro S protein/TN^T model (S protein in pale yellow, E V_{HH} in dark blue and V V_{HH} in magenta) and 7B18 PDB model (S protein in aquamarine, E V_{HH} in steel blue and V V_{HH} light pink) showing how the binding of the V_{HH}s to their respective RBDs occurs in both structures. While in two of them the positions are conserved, TN^T presence causes a shift in the third RBD location respect to the 7B18 model, even though the manner that both V_{HH}s recognize this RBD domain is preserved. For easy viewing just the three-RBDs from the S trimer are showed.

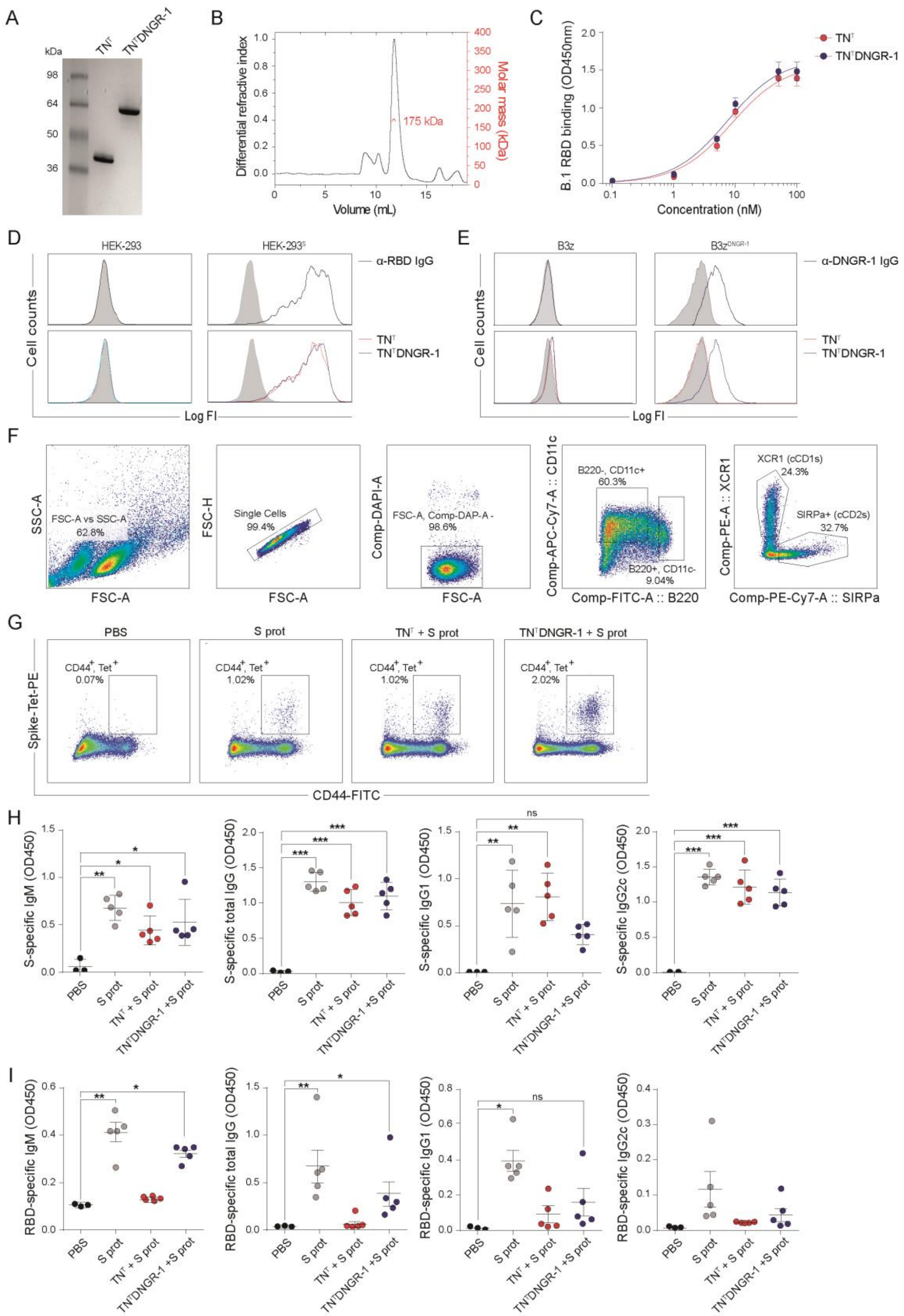

Figure S4. Purified TN^{T}DNGR-1 performs as globular bispecific trimerbody in solution targeting viral antigens to DNGR-1-expressing DCs *in vitro* and boosting systemic S-specific responses *in vivo*. A) Coomassie blue staining after SDS-PAGE analysis in reducing conditions comparing purified samples of both TN^{T} and TN^{T}DNR-

1. Each lane contains 1μg of purified protein. B) SEC-MALS analysis of TN^{T}DNR-1 showing its predominant trimeric state. The black lines correspond to the differential refractive index (left axis) and the red line to the measured molar mass (right axis). C) Comparative binding of B.1 RBD by TN^{T}DNGR-1 and parental TN^{T} by mean of ELISA assay. D) TN^{T} (red line) or TN^{T}DNGR-1 (blue line) purified proteins were incubated, 1μg/mL, with SARS-CoV-2 S protein$^{+/-}$ HEK-293 before StrepTagII detection. Histograms include anti-RBD stained (thin black line) and non-stained cells as a control (grey). E) AlexaFluor647 conjugated RBD (AF647-RBD) was added to B3z DNGR-1$^{+/-}$ cells after incubating them with TN^{T} (red line) or TN^{T}DNGR-1 (blue line). Histograms also show cells just treated with AF647-RBD (grey) and (thin black line). F) Representative gating of mouse cDC1s, cD2s and pDCs from Flt3-L BMDCs according to their surface markers staining for the analysis of TN^{T}DNGR-1 labelling assays (see Fig. 2E). Mononuclear cells isolated from mouse bone marrow were induced for differentiation *in vitro* and characterized phenotypically as follows: cDC1s (B220^{+}, CD11c^{-}, XCR1^{+}, SIRPa^{-}), cDC2s (B220^{+}, CD11c^{-}, XCR1^{-}, SIRPa^{+}) and pDCs (B220^{+}, CD11c^{-}). G) CD44^{+} S-Tet^{+} dot plot representation on gated CD8^{+} T cells corresponding to the different treatment groups (PBS, S protein + adjuvants, S protein + TN^{T} + adjuvants, S protein + TN^{T}DNGR-1 + adjuvants) of the prime-boost immunization experiment (see Fig. 2H, I). H, I) Analysis of the same prime-boost immunization experiment for S- (H) and RBD-specific (I) serum antibodies. Mice serum was collected on day 14 of the prime-boost scheme (7 days after the boost) (see Fig. 2G) and analyzed for antigen IgM, total IgG, IgG1 and IgG2c levels. Represented values correspond to samples obtained from the same experiment as the cellular immune response evaluation (n=3-5 mice/group/experiment; see Fig. 2G) using 1/50 dilution for IgM and 1/250 for total IgG, IgG1 and IgG2c.

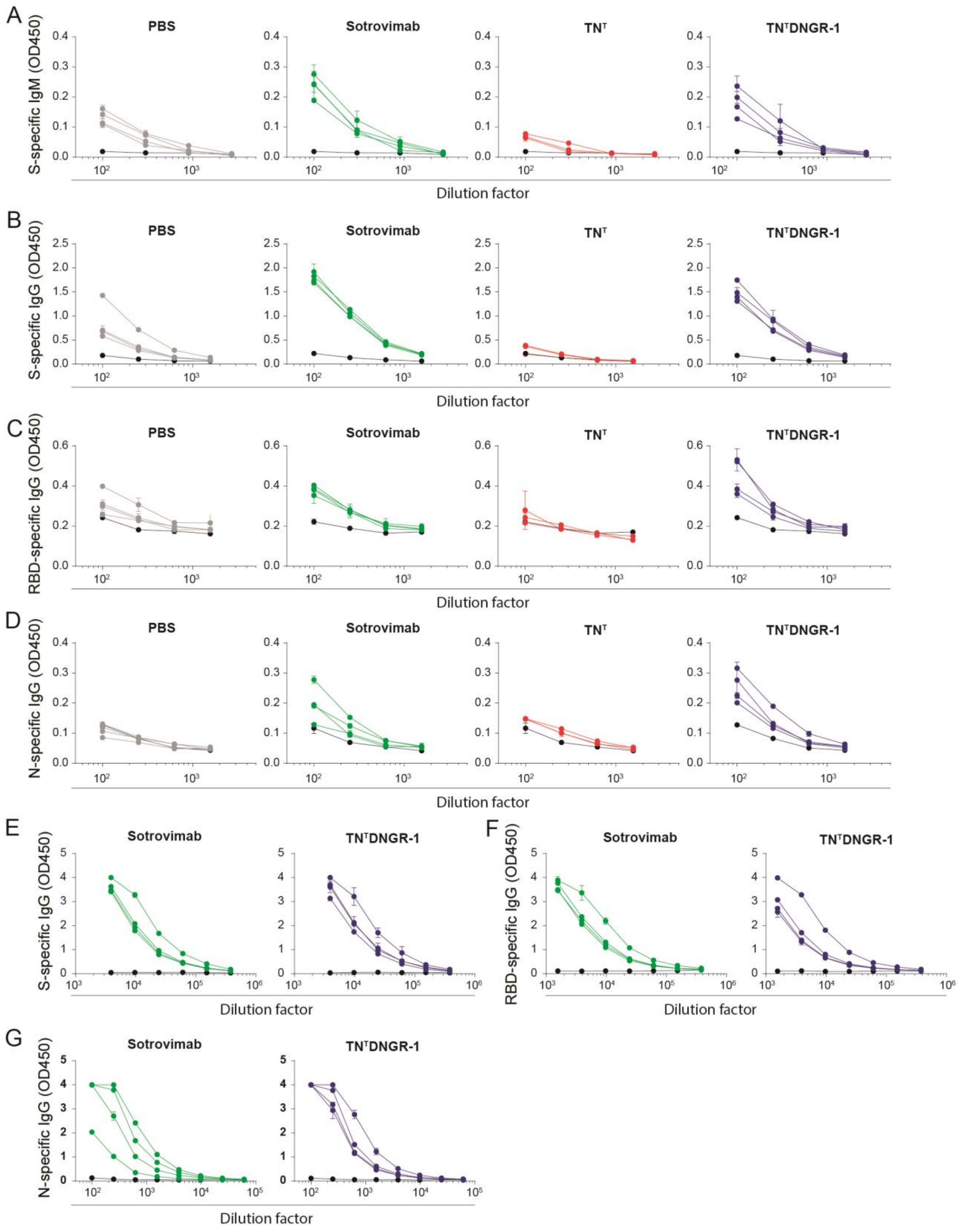

Figure S5. Absorbance measurements for serum antibodies binding to SARS-CoV-2 antigens. Data showing S-specific IgM (A), and S- (B), RBD- (C) and N-specific (D) total IgG quantification on day 5 p.c. Measurements for S- (E), RBD- (F) and N-specific (G) total IgG quantification on day 20 p.c. All graphs represent mean ± SEM (n=4

mice/group) for a single experiment. All measurements were done by ELISA by duplicate.

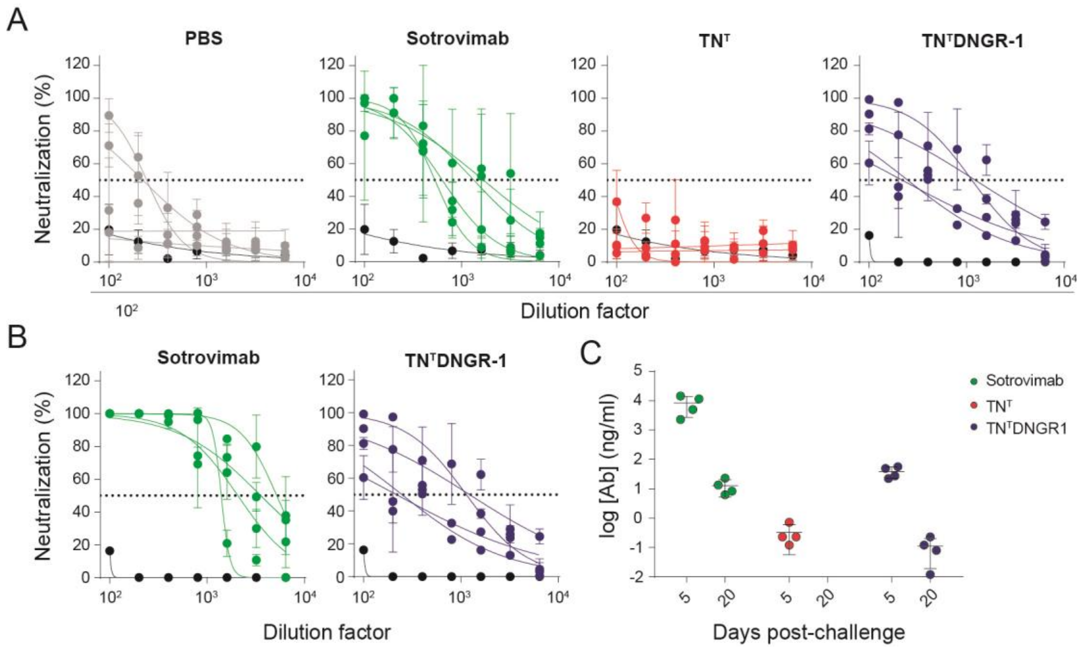

Figure S6. Efficacy experiment mice serum analysis for SARS-CoV-2 neutralization. Neutralization experiments for live SARS-CoV-2 (B.1 strain) virus using pooled mice sera from each group (n=4 mice/group) and obtained on 5 (A) and 20 (B) days p.c. NT_{50} is represented as a dotted line. C) Calculation of administrated antibodies (sotrovimab, TN^T and TN^TDNGR-1) concentration present in serum at 5- and 20-days p.c. All graphs represent mean ± SEM for a single experiment. All measurements were done by duplicate.

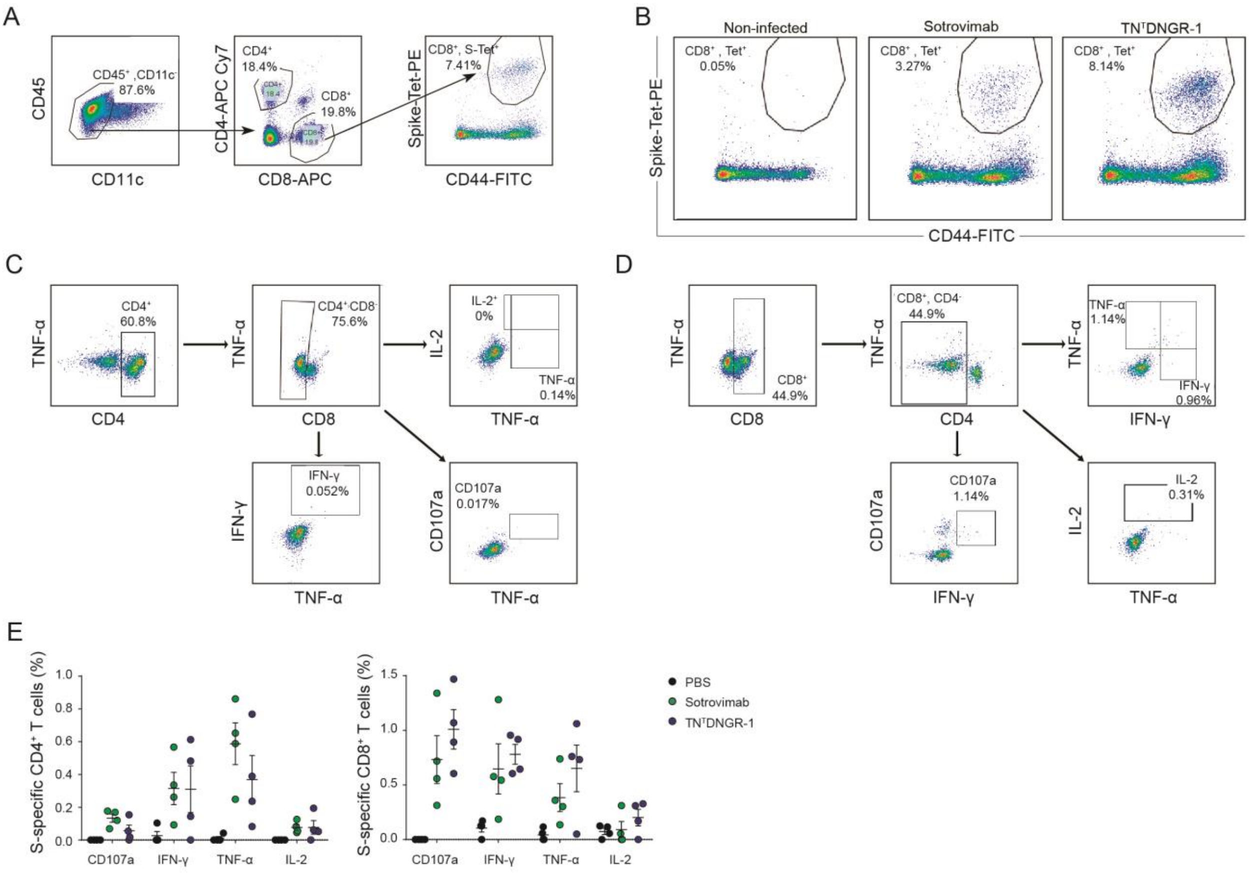

Figure S7. Flow cytometry analysis showing TN^{T}DNGR-1 treatment of SARS-CoV-2 infection induced S-specific CD8^{+} T cell responses in the lungs. A) Representative gating for S-Tet staining of CD8^{+} T cells obtained from processed K18-hACE2 mice lungs at day 20 p.c. Single and live CD8^{+} T cells were gated from lymphocyte morphology cloud as CD45^{+}, CD11c-, CD8^{+}, CD4^{-}. B) CD44^{+} S-Tet^{+} dot plot representation from processed lung CD8^{+} T cells at day 20 p.c. showing exemplary mice from the different treatment groups (PBS, sotrovimab and TN^{T}DNGR-1). For ICS data analysis CD4^{+} (C) and CD8^{+} (D) T cells were gated from single and live lymphocyte morphology cloud as CD3^{+} and CD4^{+} or CD8^{+}. Herein an exemplary ICS gating for CD107a, IFN-γ, TNF-α and IL-2 is represented as paired dot plot. E) ICS for CD107a, IFN-γ, TNF-α or IL-2, represented as frequency of CD4^{+} (left panel) and CD8^{+} (right panel) responding cells in the lungs at 20 days p.c. for each individual marker. All graphs represent individual dots and mean ± SEM (n=4 mice/group) for a single experiment.
